# Analysis of the Differentiation of Kenyon Cell Subtypes Using Three Mushroom Body-Preferential Genes during Metamorphosis in the Honeybee (*Apis mellifera* L.)

**DOI:** 10.1371/journal.pone.0157841

**Published:** 2016-06-28

**Authors:** Shota Suenami, Rajib Kumar Paul, Hideaki Takeuchi, Genta Okude, Tomoko Fujiyuki, Kenichi Shirai, Takeo Kubo

**Affiliations:** Department of Biological Sciences, Graduate School of Science, The University of Tokyo, Bunkyo-ku, Tokyo 113–0033, Japan; University of Cologne, GERMANY

## Abstract

The adult honeybee (*Apis mellifera* L.) mushroom bodies (MBs, a higher center in the insect brain) comprise four subtypes of intrinsic neurons: the class-I large-, middle-, and small-type Kenyon cells (lKCs, mKCs, and sKCs, respectively), and class-II KCs. Analysis of the differentiation of KC subtypes during metamorphosis is important for the better understanding of the roles of KC subtypes related to the honeybee behaviors. In the present study, aiming at identifying marker genes for KC subtypes, we used a cDNA microarray to comprehensively search for genes expressed in an MB-preferential manner in the honeybee brain. Among the 18 genes identified, we further analyzed three genes whose expression was enriched in the MBs: *phospholipase C epsilon* (*PLCe*), *synaptotagmin 14* (*Syt14*), and *discs large homolog 5* (*dlg5*). Quantitative reverse transcription-polymerase chain reaction analysis revealed that expression of *PLCe*, *Syt14*, and *dlg5* was more enriched in the MBs than in the other brain regions by approximately 31-, 6.8-, and 5.6-fold, respectively. *In situ* hybridization revealed that expression of both *Syt14* and *dlg5* was enriched in the lKCs but not in the mKCs and sKCs, whereas expression of *PLCe* was similar in all KC subtypes (the entire MBs) in the honeybee brain, suggesting that *Syt14* and *dlg5*, and *PLCe* are available as marker genes for the lKCs, and all KC subtypes, respectively. *In situ* hybridization revealed that expression of *PLCe* is already detectable in the class-II KCs at the larval fifth instar feeding stage, indicating that *PLCe* expression is a characteristic common to the larval and adult MBs. In contrast, expression of both *Syt14* and *dlg5* became detectable at the day three pupa, indicating that *Syt14* and *dlg5* expressions are characteristic to the late pupal and adult MBs and the lKC specific molecular characteristics are established during the late pupal stages.

## Introduction

The European honeybee (*Apis mellifera* L.) is a social insect and colony members exhibit various exquisite social behaviors. Female adults differentiate into queens (reproductive caste) and workers (labor caste), and the workers shift their labors from nursing their brood (nurse bees) to foraging for nectar and pollen (foragers), associated with the age after eclosion [1–3]. In addition, foragers that return from foraging flights communicate the location of food sources to their nestmates using ‘dance language’ [3, 4]. These characteristics make the honeybee an excellent model animal for study of the neural bases underlying the highly advanced social behaviors.

The brains of insects including the honeybee comprise the optic lobes (OLs, a visual center), antennal lobes (ALs, an olfactory center), and mushroom bodies (MBs). The insect MBs are a paired structure that acts as the higher center for sensory information processing, learning, and memory, and comprise intrinsic neurons called Kenyon cells (KCs) [5–8]. In the honeybee, each MB has two calyces and the KC somata are located inside (class-I KCs) and at the periphery of the calyces [(clawed) class-II KCs or ‘outer-compact’ KCs]. The class-I KCs are further classified into three subtypes: large- (lKCs or ‘noncompact’ KCs), middle- (mKCs) and small-type (sKCs or ‘inner-compact’ KCs), based on the localization and size of their somata [5, 6, 9, 10, 11]. The somata of lKCs are located at the inside edges of the calyces, whereas the somata of sKCs are located at the innercore of the calyces [5, 6, 9, 10]. In addition, we recently identified a novel KC subtype, which we termed mKCs that are characterized with the preferential expression of *mKast* (*m**iddle-type* *K**C preferential* *a**rre**st**in-related protein*) [11]. The somata of mKCs are localized sandwiched between those of lKCs and sKCs [11]. The lKCs and sKCs have been shown to have distinct dendritic projections in the MB calyces and functions in sensory information processing [5, 6, 9, 10].

In *Drosophila*, genetic studies revealed that the MBs are involved in olfactory learning. Some genes involved in olfactory learning: *dunce*, *rutabaga*, and *DCO*, which encode cAMP phosphodiesterase, a type I calcium/calmodulin-stimulated adenylate cyclase, and catalytic subunit of protein kinase A (PKA), respectively, are predominantly expressed in the MBs, demonstrating the role of cAMP-signaling in learning and memory in *Drosophila* [12–15]. In the honeybee, the MBs are involved in learning and memory [16–18], and in higher-order multimodal computations [19, 20]. The expression of the gene for catalytic subunit of PKA is enriched in the MBs [21], and phosphorylated cAMP-response element binding protein is also predominantly localized in the sKCs in the honeybee [22].

In the honeybee, each KC subtype has distinct gene expression profile, suggesting their distinct functions; to date, however, no single study has investigated the gene expression and dendritic projections of these subtypes, and thus their distinct functions remain an assumption. Expression of some genes involved in calcium signaling, such as *inositol 1*,*4*,*5-trisphosphate receptor* (*IP*_*3*_*R*) [23], *type I IP*_*3*_ *5-phosphatase* [24], *calcium/calmodulin-dependent protein kinase II* (*CaMKII*) [25], *ryanodine receptor* (*ryr*), and *reticulocalbin* [26], is enriched in the lKCs, suggesting that the function of calcium signaling [27] is enhanced in the lKCs in the honeybee brain. In contrast, expression of some genes involved in ecdysone signaling, such as *ecdysone receptor* (*EcR*) [28], *HR38* [29] and *E74* [30] is enriched in the sKCs. In addition, expression of *kakusei*, an immediate early gene, is expressed mainly in the sKCs and a part of the mKCs in the forager brains, suggesting the roles of sKCs and mKCs in information processing during the foraging flight [11, 31].

In the honeybee, the larval and adult MBs comprise distinct KC subtypes. Farris et al. (1999) reported that there are only the class-II KCs in the larval MBs and the class-I lKCs and sKCs are sequentially generated from the MB neuroblasts in the pupal brain during metamorphosis based on the morphological observation [32]. However, as there have been no marker genes for KC subtypes, it remained unclear whether the larval and adult class-II KCs share the same molecular characteristics and when each KC subtype starts to differentiate during metamorphosis. As the larval and adult behaviors completely differ in the honeybee [1, 32], we expected that the analysis and comparison of the marker gene expression in the larval, pupal and adult MBs could provide important insights into the roles of KC subtypes related to the honeybee behaviors.

In the present study, we used a cDNA microarray to comprehensively search for genes whose expression is enriched in the adult honeybee MBs, and identified three genes, *phospholipase C epsilon* (*PLCe*), and *synaptotagmin 14* (*Syt14*) and *discs large homolog 5* (*dlg5*), that are available as marker genes for the entire MBs and lKCs, respectively. Expression analysis of these genes revealed that preferential *PLCe* expression is a characteristic common to the larval and adult MBs, whereas preferential expression of *Syt14* and *dlg5* is unique to the adult MBs.

## Materials and Methods

### Animals

European honeybee colonies maintained at the University of Tokyo were used. Nurse bees and foragers were collected from the hives. Worker bees in-hive and with well-developed hypopharyngeal glands, and bees with pollen loads on their hind legs and regressed hypopharyngeal glands were collected as nurse bees and foragers, respectively, as described previously [11]. Worker bees patrolling at the hive entrance and the other in-hive bees were collected as aggressive and non-aggressive workers, respectively [24, 29].

### cDNA microarray

In the present study, we used a cDNA microarray, which we previously prepared to identify genes expressed in the honeybee brain in an OL-preferential manner [11, 33] or in a role-dependent manner [24, 29], to compare gene expression profiles between the MBs and OLs as well as between the MBs and ALs. For this, total RNA was extracted from the MBs, OLs and ALs of approximately 1000 workers and then poly (A)+ RNA was isolated using Oligotex-dT30 Super (TaKaRa). 2μg of the poly (A)+RNA was reverse-transcribed with random primers and the resulting cDNA was labeled with Cy3 (for cDNA from the MBs) or Cy5 (for cDNAs either from the OLs or ALs) using Atlas Powerscript Fluorescent Labeling Kit (Clontech). Cy3- and Cy5-labeled probes were mixed and purified using Microcon-30 (Millipore). Hybridization and washing were performed as described previously [11, 24, 29, 33]. Expression level of each clone was measured using Scan Array 4000 (GSI Lumonics), normalized with that of *β-actin* expression in each tissue, and the fluorescence ratio between Cy3 and Cy5 was quantified. Hybridization was duplicated with the same RNA preparations. When higher expression of a cDNA clone was reproducibly detected in the MBs than in the OLs or ALs by 2.0-folds or more, the clone was assigned as positive, and its expression was further confirmed by *in situ* hybridization. When only weak signal was detected in the MBs or strong signal was detected in the other brain regions by *in situ* hybridization, the clone was assigned as negative and remained clones were further characterized.

### Cloning of cDNAs and preparation of RNA probes

**Fig 1:**
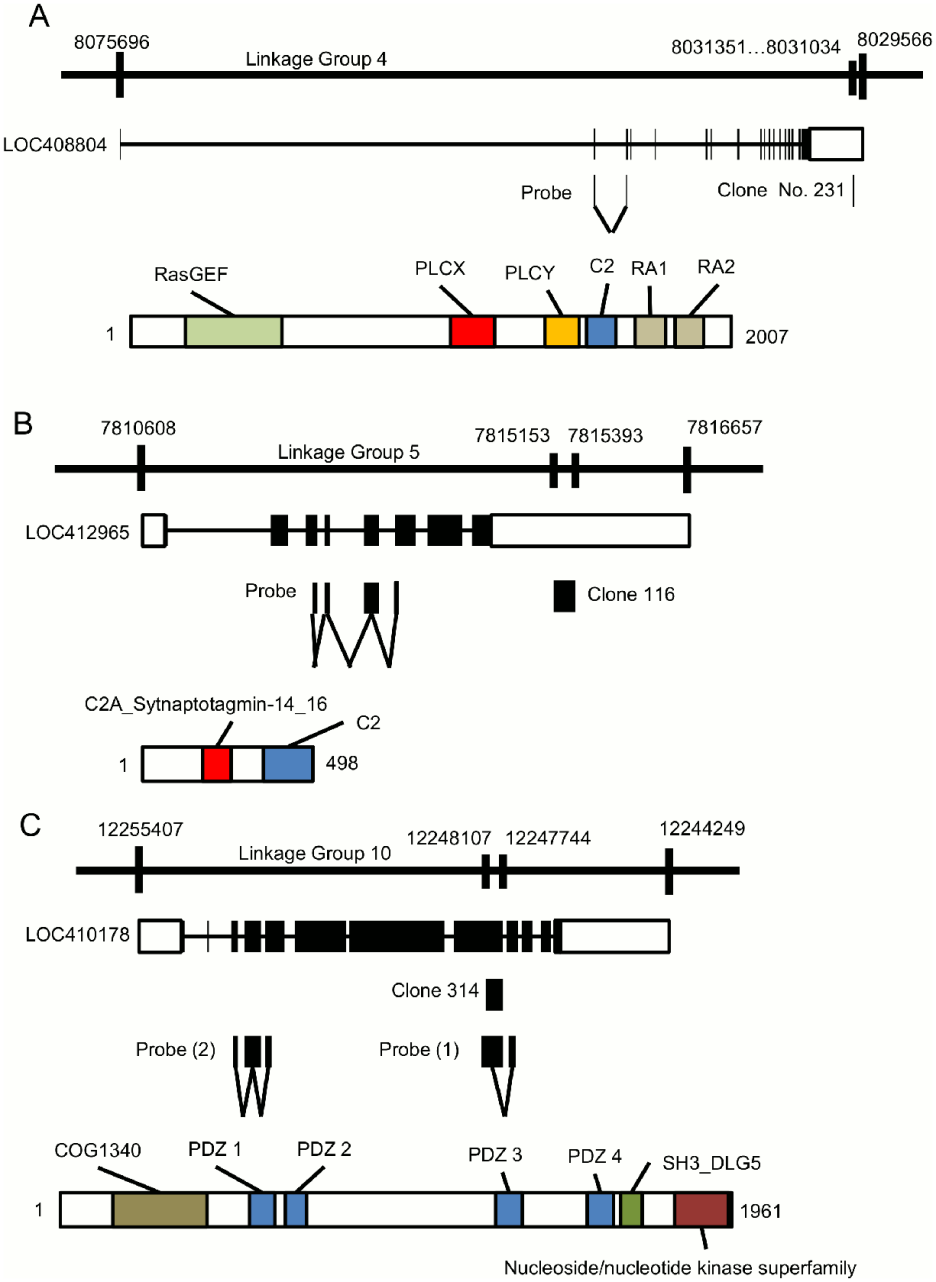
Characteristics of identified honeybee genes showing preferential MB expression. The gene and protein structures of (A) *PLCe*, (B) *Syt14*, and (C) *dlg5*. In each panel, the upper line indicates linkage group where the corresponding gene is mapped. The second line indicates the predicted gene structure. Closed boxes indicate coding regions, while open boxes indicate 5’ and 3’ UTRs. Positions of differential display products (clone Nos. 231, 116, and 314, respectively) and probes used for *in situ* hybridization are shown below the gene structure. The lower bar indicates protein structure. Numbers indicate amino acid positions. Colored boxes on the protein structure show functional domains.

Total RNA extracted from the brains of adult workers was treated with DNase I Amp Grade (Invitrogen) and reverse-transcribed using SuperScript III (Invitrogen) with random primers. The cDNA was partially amplified by polymerase chain reaction (PCR) using Ex *Taq* (Takara) with the following gene specific primers, which were designed based on the predicted mRNA deposited in the Genbank (accession numbers are shown in parentheses): *PLCe*, 5’-GTTTCGCCAATCGAAAAACG-3’ and 5’-CGAATACCAGCTGTTCTACC-3’ (XM_392335.4); *Syt14*, 5’-TCCACCGCCGGATACTTTAAC-3’ and 5’-CCAGGGTACATCTCCACAATACA-3’ (XM_396416.4); *dlg5*, two pairs of primer sets were used. The first pair represented as (1) in Fig 1C was 5’-GTGCTCGAATCTAGGCATCTC-3’ and 5’-CGTTCTTCCAGCAGAGATAGT-3’, and the second pair represented as (2) in Fig 1C was 5’- CTTAGGCATAAACACTCCGATAC-3’ and 5’-GTTGCTTCAAACCATTACGCTC-3’ (XM_393661.4); *mKast*, 5’-GCAGCTTCGAGGGCCGATAC-3’ and 5’-TAGTACCACTCGCTCACGTC-3’ (XM_001121375.3); *Ribosomal protein L32* (*RpL32*), 5’-TCGTCACCAGAGTGATCGTT-3’ and 5’-CGTAACCTTGCACTGGCATT-3’ (XM_006564315.1); and *Mblk-1*, 5’-AATTTCAAATTTCGCCTCGA-3’ and 5’-TTTTGGAACAACCCCACCAT-3’ (AB047034.1). The PCR products were cloned using pGEM-T Easy (Promega). PCR was performed to obtain templates for *in vitro* transcription using Takara Ex *Taq* with M13 forward and reverse primers. The DIG-labeled sense and antisense RNA probes were prepared by *in vitro* transcription using a DIG labeling Kit (Roche) as described previously [11, 29, 33]. The lengths of the resultant probes are as follows: *PLCe*; 425bp, *Syt14*; 410bp, *dlg5*; (1) 580bp and (2) 484bp, *mKast*; 526bp, *RpL32*; 327bp, *Mblk-1*; 273bp.

### Multiple alignment

Some EST and transcriptome sequence assembly (TSA) sequences available from the NCBI database were used to identify the full-length cDNA structures of *PLCe*, *Syt14*, and *dlg5*. All of the predicted cDNA sequences were covered by the EST and TSA data, except one exon-intron junction in *PLCe*. The sequence of this junction was subcloned by a set of primers (+1 to +20, 5’-ATGTGCGAGGACAATAAAGC -3’; and +482 to +501, 5’-CACAAGACACGCAGAAACTA-3’). When sequencing the corresponding cDNA fragment, the subcloned sequence coincided with that registered in the NCBI genome database (Genbank: XM_392335.4), and so this sequence was used for the further analysis.

Proteins and their accession numbers for PLCe, Syt14 and dlg5 homologs from the honeybee, fruit fly, red flour beetle (*Tribolium castaneum*), yellow fever mosquito (*Aedes aegypti*), southern house mosquito (*Culex quinquefasciatus*), nematode (*Caenorhabditis elegans*), house mouse (*Mus musculus*), and human used in the sequence alignment are summarized in a S4 Table. Alignment was performed using ClustalW and identities were calculated using BioEdit software (http://www.mbio.ncsu.edu/bioedit/bioedit.html).

### Quantitative reverse transcription (RT)-PCR analysis

Adult workers were randomly collected from the hive. After the bees were anesthetized on ice, the MBs and the other brain regions were dissected from their heads with fine tweezers and scalpels under binocular microscope. Briefly, first, connections between the OLs and retina, and other connections between the whole brains and the surrounding trachea or muscle were removed, and the whole brains were dissected out from the heads. If ocelli were attached to the MBs, they were removed using tweezers. Then, connections between the MBs and OLs, and next connections between the MBs and ALs were removed by scalpels, and the MBs were retrieved. One lot contained two workers, and four lots were used for the experiment. For the gene expression analysis in the larval and pupal brains, brains were dissected from the fifth instar worker larvae at the feeding stage (L5F) and worker pupae 2, 3, 7 and 9 days after puparium formation (P2, P3, P7, and P9, respectively). The larval and pupal stages were determined according to the previous reports [34, 35]. Five (L5F), three (P2), two (P3) and one (P7 and P9) bees were used as a lot, respectively, and three lots of samples were analyzed for each stage. The brains were homogenized in TRIzol LS Reagent (Ambion) soon after the dissection and stored at -80°C until use.

After homogenizing the brain samples, total RNA was extracted from the samples and reverse-transcribed with PrimeScript RT reagent Kit with gDNA eraser (Perfect Reat Time) (TaKaRa). Quantitative RT-PCR was performed with LightCycler and LightCycler 480 Instrument II (Roche) using SYBR Premix Ex Taq II (Tli RNaseH Plus) (TaKaRa) essentially as described previously [36]. The primers used are as follows: *PLCe*, 5’-GTTTCGCCAATCGAAAAACG-3’ and 5-GATAGAGGTCAATGGAGCCA-3’; *Syt14*, 5’-TCCACCGCCGGATACTTTAAC-3’ and 5’-CAATGCGTGGTAGAAGTGGG-3’; *dlg5*, 5’-GTGCTCGAATCTAGGCATCTC- 3’ and 5’-CGTTGATCTCAGACAGGCAA-3’; *Mblk-1*, 5’-CAACACCAAATACGACCCAAAAC-3’ and 5’-GACAACAGCGGCTTCAAC-3’; *RpL32*, 5’-AAAGAGAAACTGGCGTAAACC-3’ and 5’-CTCGTCATATGTTGCCAACTG-3’; *glyceraldehyde 3-phosphate dehydrogenase* (*gapdh*, XM_393605.5), 5’-GATGCACCCATGTTTGTTTG-3’ and 5’-TTTGCAGAAGGTGCATCAAC-3’. *RpL32* and *gapdh* were quantified as reference genes [37, 38]. The amount of transcripts of *PLCe*, *Syt14*, *dlg5* and *Mblk-1* was normalized to that of the *RpL32* transcript. Welch’s t-test and Kruskal-Wallis test were performed for statistical analysis in the adult and developing brains, respectively.

### *In situ* hybridization analysis

For *in situ* hybridization, the dissected bee brains were embedded in Tissue-Tek OCT compound (Sakura Finetek Japan) without fixation and frozen on dry ice. *In situ* hybridization was performed essentially as described previously [11, 33, 36]. The sections (10 μm thick) were fixed with 4% paraformaldehyde in 0.1 M phosphate buffer (4% PFA-PB) (pH 7.4) overnight. They were treated with proteinase K (10 μg/ml) for 15 min, 4% PFA-PB again for 15min, 0.2 M HCl for 10 min, and 0.25% acetic-anhydride in 0.1 M tri-ethanolamine HCl for 10 min. The slides were washed with phosphate buffer between each step. All of these treatment steps were performed at room temperature. The tissue was dehydrated in an ascending ethanol series (from 70% to 100%, increasing by 10%), and the sections were hybridized with RNA probes. The probes were diluted in hybridization buffer (50% formamide, 10 mM Tris-HCl at pH 7.6, 200 μg/ml tRNA, 1x Denhardt’s solution, 10% dextran sulfate, 600 mM NaCl, 0.25% SDS, 1 mM EDTA) before use. The hybridization condition was: overnight (>12 h) at 50°C. After washing with 50% formamide in 2x saline sodium citrate (SSC) for 1 h at 50°C, sections were treated with TNE (10 mM Tris-HCl at pH 7.5, 0.5 M NaCl, 1 mM EDTA) for 10 min at 37°C. RNase A was added to the TNE (final concentration of RNase A was 10 μg/ml) and the slides were treated for 15 min at 37°C. The slides were then washed in fresh TNE for 10 min at the same temperature, and then in 2x SSC for 20 min at 50°C and twice in 0.2x SSC for 20 min at 50°C. The probes were detected using a DIG Nucleic Acid Detection Kit (Roche). The slides were treated with DIG buffer I (100 mM Tris-HCl pH 7.5, 150 mM NaCl) for 5 min, 1.5% blocking reagent (Roche) for 1 h, and anti-DIG antibody conjugated with alkaline phosphatase (1:1000; Roche) for 30 min. After washing in DIG buffer I twice for 15 min, the sections were treated with DIG buffer III (100 mM Tris-HCl pH 9.5, 100 mM NaCl, 50 mM MgCl_2_, and 0.005% Tween 20) for 3 min. All steps were performed at 25°C. NBT/BCIP stock solution (Roche) was diluted (1:50) and mounted on sections for detection of the probes. The staining reactions were performed at 25°C for 3 h (*PLCe*), 6 h (*RpL32*), and >12 h (the other genes). Image reconstruction was processed using Photoshop software (Adobe Systems) and GIMP2.8 software (http://www.gimp.org/).

*In situ* hybridization using probes targeting cDNA clones was performed for two or three adult brains for each clone, while *in situ* hybridization targeting coding regions of *PLCe*, *Syt14*, and *dlg5* was performed using two larval, two pupal, and more than two adult brains for each gene, to confirm the reproducibility of the results.

## Results

### Comprehensive search for genes expressed preferentially in the honeybee MBs

We previously used a combination of the differential display method and cDNA microarray to search for genes expressed in an OL-preferential manner [11, 33] or in a role-dependent manner [24, 29] in the honeybee brain. In the present study, aiming at identifying marker genes for KC subtypes, we used the same cDNA microarray to comprehensively search for genes that are expressed in an MB-preferential manner in the honeybee brain. Hybridization with mixture of Cy3-labeled probe derived from the MB RNA, and Cy5-labeled probes derived from the OL or AL RNAs, yielded 112 positive clones. When expression level of a certain cDNA clone was higher in the MBs than in the OLs or ALs at least by 2.0-folds, *in situ* hybridization was performed to analyze its expression in the brains. In most cases, signals of expression in the MBs or in the other brain regions were detected evenly. Finally, 18 clones were identified as expressed in an MB-preferential manner (S1 Table and S1 Fig). Next, genes, on which the identified cDNA fragments were mapped, were identified using the NCBI genome database (S2 Table). When the cDNA clones were mapped onto two genes or intergenic regions, we annotated that corresponding gene as “not identified” and listed the two genes that flank those clones in the honeybee genome (S2 Table). Nucleotide sequences for clones except clone Nos. 60 and 495 (S3 Table) are registered to NCBI database.

Among the 18 cDNA fragments identified, four (clone Nos. 105, 116, 231, 314) corresponded to predicted genes for proteins related to intracellular signal transduction, two (clone Nos. 440 and 567) corresponded to those for transcription factors, one (clone No. 60) to that for a cell-cell adhesion molecule, one (clone No. 28) to that for neurotransmitter receptor, two for non-coding RNAs (clone Nos. 9 and 539). One (clone No. 299) corresponded to a gene for a protein whose function is unknown. For five clones (clone Nos. 302, 443, 463, 466, and 495), corresponding genes could not be identified, because the clones were mapped onto intergenic regions. One clone (clone No. 387) was mapped onto two overlapping genes while another clone (clone No. 523) was not flanked by other genes, thus corresponding genes were unidentifiable for these two clones (S2 Table).

*In situ* hybridization analysis using the differential display fragments as probes suggested that seven (clone Nos. 116, 299, 314, 387, 443, 463, and 466) were preferentially expressed in an lKC-preferential manner, whereas one (clone No. 567) was preferentially expressed in both the mKCs and sKCs, three (clone Nos. 105, 440 and 495) were preferentially expressed in the mKCs, sKCs, and class-II KCs (i.e., not or weakly expressed in the lKCs). Two clones (clone Nos. 28 and 539) were expressed preferentially in both the lKCs and sKCs. Five clones (clone Nos. 9, 60, 231, 302, and 523) were expressed in the whole MBs (all KC subtypes; S1 Fig and S2 Table). These findings support the notion that each KC subtype has a distinct, yet overlapping gene expression profile.

### Annotation of three genes expressed in the honeybee MBs

**Fig 2:**
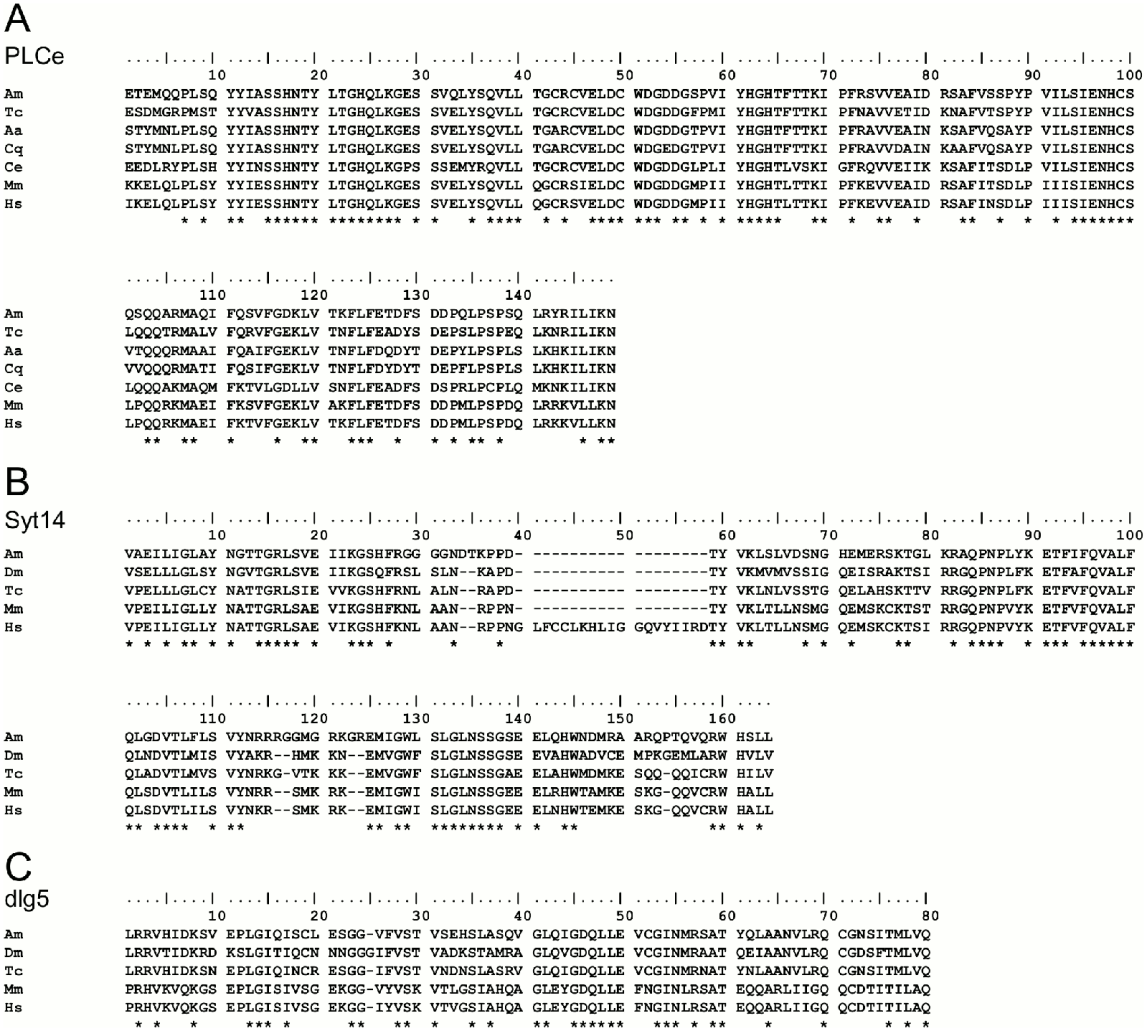
Alignment of amino acid sequences of PLCe, Syt14, and dlg5. Alignments of domains showing the highest similarity in each protein with other known protein orthologues. (A) PLCX catalytic domain of PLCe, (B) C2 of Syt14 and Syt16, (C) The third PDZ domain of dlg5. Amino acid sequence of honeybee PLCe was deduced from the nucleotide sequence of GB42984. Alignments were performed using ClustalW. Identical amino acids in the same position are labelled by asterisks under the alignments. Abbreviations of species are follows: Am, *Apis mellifera*; Dm, *Drosophila melanogaster*; Tc, *Tribolium castaneum*; Aa, *Aedes aegypti*; Cq, *Culex quinquefasciatus*; Ce, *Caenorhabditis elegans*; Mm, *Mus musculus*; Hs, *Homo sapiens*. Some species are not used in each alignment because their proteins or domains are not annotated.

Among the 18 cDNA clones identified, we focused on three (clone Nos. 116, 231, and 314) whose signals were detected almost exclusively in the MBs or certain KC subtypes for the subsequent expression analysis. They were mapped the onto coding region or 3’ untranslated region (3’ UTR), making it easy to identify corresponding candidate genes. Expression of the other 15 clones was either partially detected in the other brain regions than the MBs, such as the OLs and ALs, or expression in the MBs was not very strong (S1 Fig and S1 Table). Database searches revealed that clone No. 231 (GenBank: BP539402) is located on 3’ UTR of LOC408804 (Fig 1A), which encodes a predicted protein that was most similar to phospholipase C epsilons (PLCes). Alignment of the amino acid sequence of the LOC408804 product with those of *Tribolium castaneum* (red flour beetle), *Aedes aegypti* (yellow fever mosquito), *Culex quinquefasciatus* (southern house mosquito), *Caenorhabditis elegans* (nematode), *Mus musculus* (mouse), and human revealed 45.4%, 23.7%, 36.3%, 27.4%, 23.2%, and 23.5% overall sequence identity, respectively. Alignment of the domains indicated that the catalytic domains of PLC [39] are highly conserved among species (Fig 2A and S5A Table). Based on these findings, we concluded that LOC408804 encodes a honeybee homolog of PLCe.

Clone No. 116 (GenBank: AB811436) was located on the 3’ UTR of LOC412965, which is predicted to be *synaptotagmin 14* (*Syt14*) (Fig 1B). The amino acid sequence of the LOC412965 product was compared with homologs of synaptotagmins of other animals. From *Tribolium castaneum*, amino acid sequence of Syt16 was aligned, because *Syt14* and *Syt16* are synonymous in the honeybee. LOC412965 showed significant sequence identities with Syt14 of other animal species with overall identity ranging from 26.0% to 38.4% (Fig 2B and S5B Table). We therefore concluded that LOC412965 encodes a honeybee homolog of Syt14.

Clone 314 (GenBank: BP539538) was mapped to an exon of LOC410178, annotated as *discs large homolog 5-like* (*dlg5*), which is a member of membrane-associated guanylate kinase family (Fig 1C). Multiple alignments revealed an overall amino acid sequence identity ranging from 23.0 to 39.7% (Fig 2C and S5C Table). Although PDZ1-4 domains were highly conserved among the dlg5 homologs, PDZ 3 and 4 domains had higher identity than PDZ 1 and 2 domains (Fig 2C and S5C Table). From these, we concluded that LOC410178 encodes a honeybee dlg5 homolog.

### Expression analysis of the three genes in the adult worker brain

**Fig 3:**
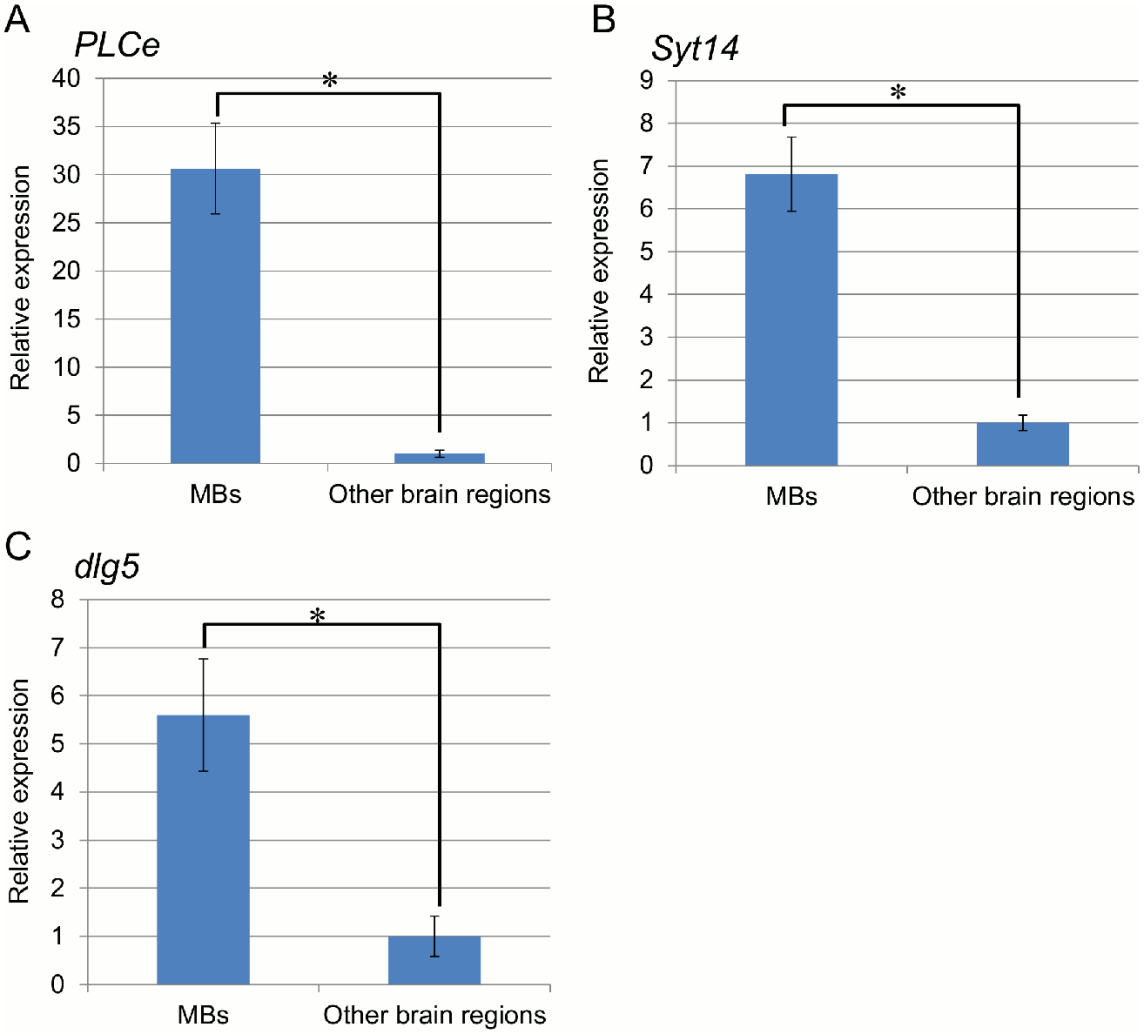
Quantitative RT-PCR analysis of *PLCe*, *Syt14*, and *dlg5*. (A) *PLCe* (B) *Syt14* (C) *dlg5*. Relative expression of each gene was normalized to *RpL32* and compared between the MBs and other brain parts, taking expression in the other brain region as 1. One lot contained two workers, and four lots were used for the experiment. Bars indicate standard deviation. * indicates *P* < 0.01 (Welch’s t-test).

Next, to confirm an MB-preferential expression of the three genes, we performed quantitative RT-PCR and *in situ* hybridization analysis. Clone Nos. 231 and 116 were located on 3’ UTR of LOC408804 (Fig 1A) and LOC412965 (Fig 1B), respectively. Therefore, we analyzed expression of the open reading frames of these genes. Quantitative RT-PCR analysis showed that the relative expression levels of *PLCe*, *Syt14*, and *dlg5* are higher in the MBs by approximately 31-, 6.8-, and 5.6-fold, respectively, than those in the other brain regions (Fig 3, Welch’s t-test, P< 0.01). Expression of *RpL32*, which was used to normalize the expression levels of these three genes, in the MBs was approximately 50% of that in the other brain regions (S2 Fig).

**Fig 4:**
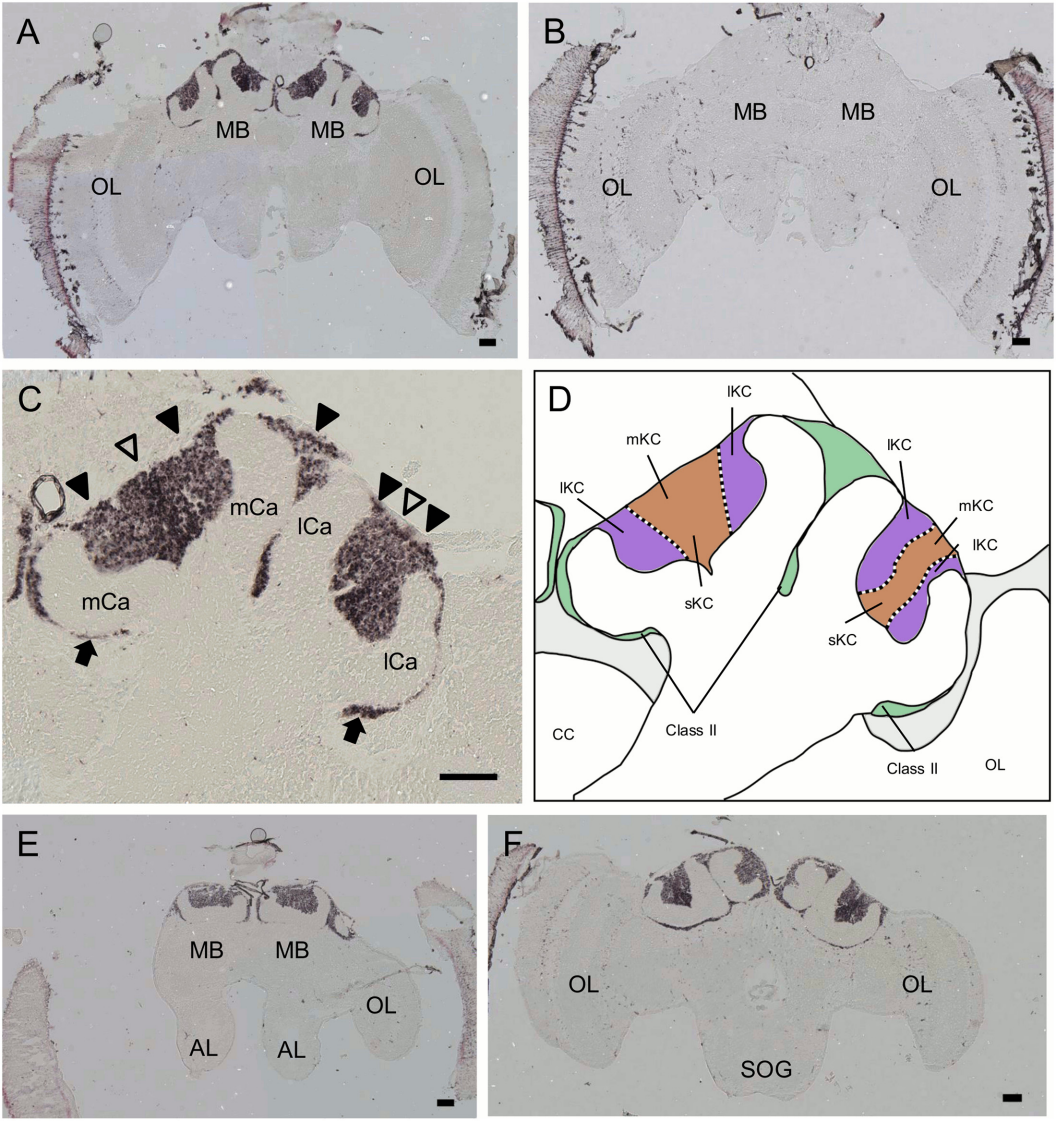
*In situ* hybridization of *PLCe* in the worker brain. (A, B) Frontal section of the whole brain hybridized with antisense (A) and sense (B) probes. (C) Magnified view of the right MB shown in (A). Filled triangles, open triangles and arrows indicate the lKCs, mKCs and sKCs, and class-II KCs, respectively. All KC types strongly expressed *PLCe*. Note that regions where the somata of mKCs are localized are not discriminated in this experiment. (D) Schematic view of (C). Boundary between the lKCs (purple), and mKCs and sKCs (orange) are indicated by dotted lines. Brain regions where the somata of the class-II KCs and other neurons exist are colored by green and light grey, respectively. (E, F) Anterior section, which contained the ALs, and posterior section, which contained the SOG, of (A) hybridized with antisense probes, respectively. MB, mushroom body; OL, optic lobe; mCa, medial calyx; lCa, lateral calyx; AL, antennal lobe; SOG, suboesophageal ganglion. Bars indicate 100 μm.

**Fig 5:**
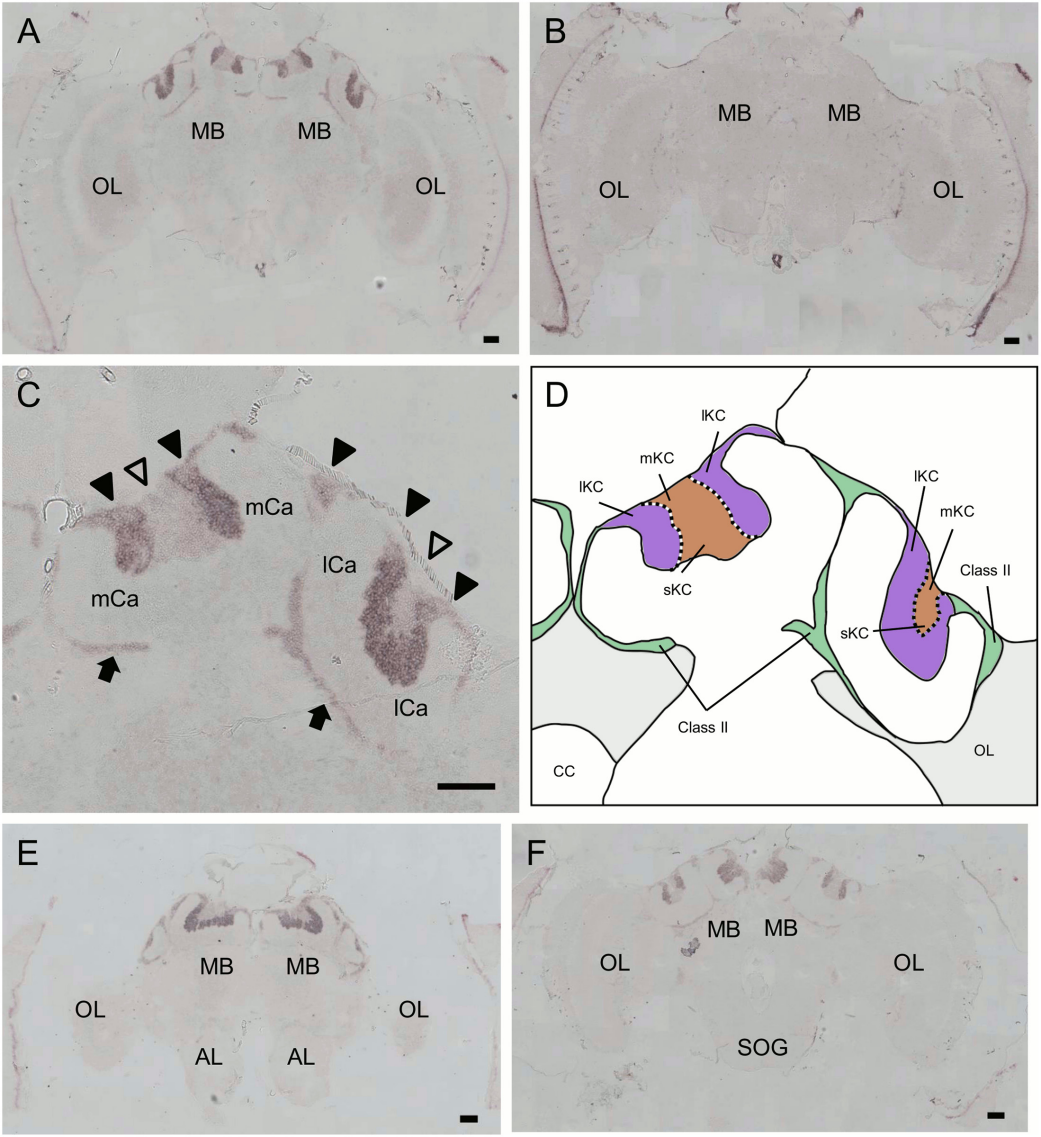
*In situ* hybridization of *Syt14* in the worker brain. (A, B) Frontal section of the whole brain hybridized with antisense (A) and sense (B) probes. (C) Magnified view of the right MB shown in (A). Filled triangles, open triangles and arrows indicate the lKCs, mKCs and sKCs, and class-II KCs, respectively. The lKCs strongly expressed *Syt14*. Note that regions where the somata of mKCs are localized are not discriminated in this experiment. (D) Schematic view of (C). Boundary between the lKCs (purple), and mKCs and sKCs (orange) are indicated by dotted lines. Brain regions where the somata of the class-II KCs and other neurons exist are colored by green and light grey, respectively. (E, F) Anterior section, which contained the ALs, and posterior section, which contained the SOG, of (A) hybridized with antisense probes, respectively. MB, mushroom body; OL, optic lobe; mCa, medial calyx; lCa, lateral calyx; AL, antennal lobe; SOG, suboesophageal ganglion. Bars indicate 100 μm.

**Fig 6:**
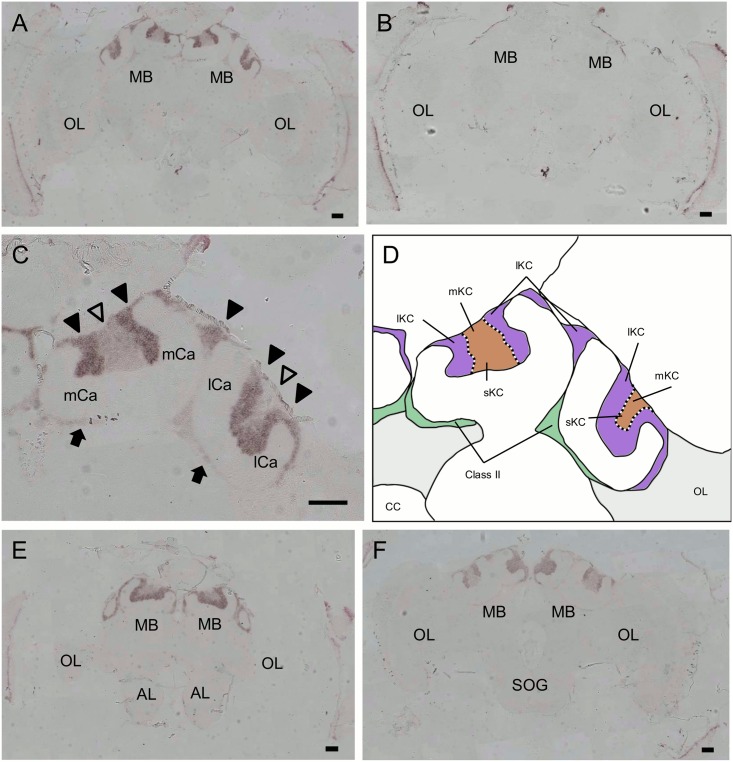
*In situ* hybridization of *dlg5* in the worker brain. (A, B) Frontal section of the whole brain hybridized with antisense (A) and sense (B) probes. (C) Magnified view of the right MB shown in (A). Filled triangles, open triangles and arrows indicate the lKCs, mKCs and sKCs, and class-II KCs, respectively. The lKCs strongly expressed *dlg5*. Note that regions where the somata of mKCs are localized are not discriminated in this experiment. (D) Schematic view of (C). Boundary between the lKCs (purple), and mKCs and sKCs (orange) are indicated by dotted lines. Brain regions where the somata of the class-II KCs and other neurons exist are colored by green and light grey, respectively. (E, F) Anterior section, which contained the ALs, and posterior section, which contained the SOG, of (A) hybridized with antisense probes, respectively. MB, mushroom body; OL, optic lobe; mCa, medial calyx; lCa, lateral calyx; AL, antennal lobe; SOG, suboesophageal ganglion. Bars indicate 100 μm. The probes were prepared with the first set of primers mentioned in Materials and Methods.

We next used *in situ* hybridization to confirm that these genes were preferentially expressed in the MBs with probes corresponding to the open reading frames. Although clone No. 314 was mapped to an exon of LOC410178 (Fig 1C), we performed *in situ* hybridization using a newly designed probe spanning multiple exons, because the probe that we used for the first *in situ* hybridization corresponded to a differential display fragment and was rather short. Strong expression of *PLCe* was detected in the somata of all of the class-I (lKCs, mKCs and sKCs) and class-II KCs (Fig 4). No significant signal was detected in the other brain regions, such as the OLs, ALs, and suboesophageal ganglion (SOG). In contrast, expression of *Syt14* and *dlg5* was stronger in the lKCs than in the other KC subtypes (*Syt14*, Fig 5; *dlg5*, Fig 6). No significant expression of *Syt14* or *dlg5* was detected in the other brain regions, consistent with the findings obtained using probes corresponding to the differential display products (S1 Fig). The expression patterns of all of these three genes did not differ significantly between the brains of nurse bees and foragers (data not shown).

**Fig 7:**
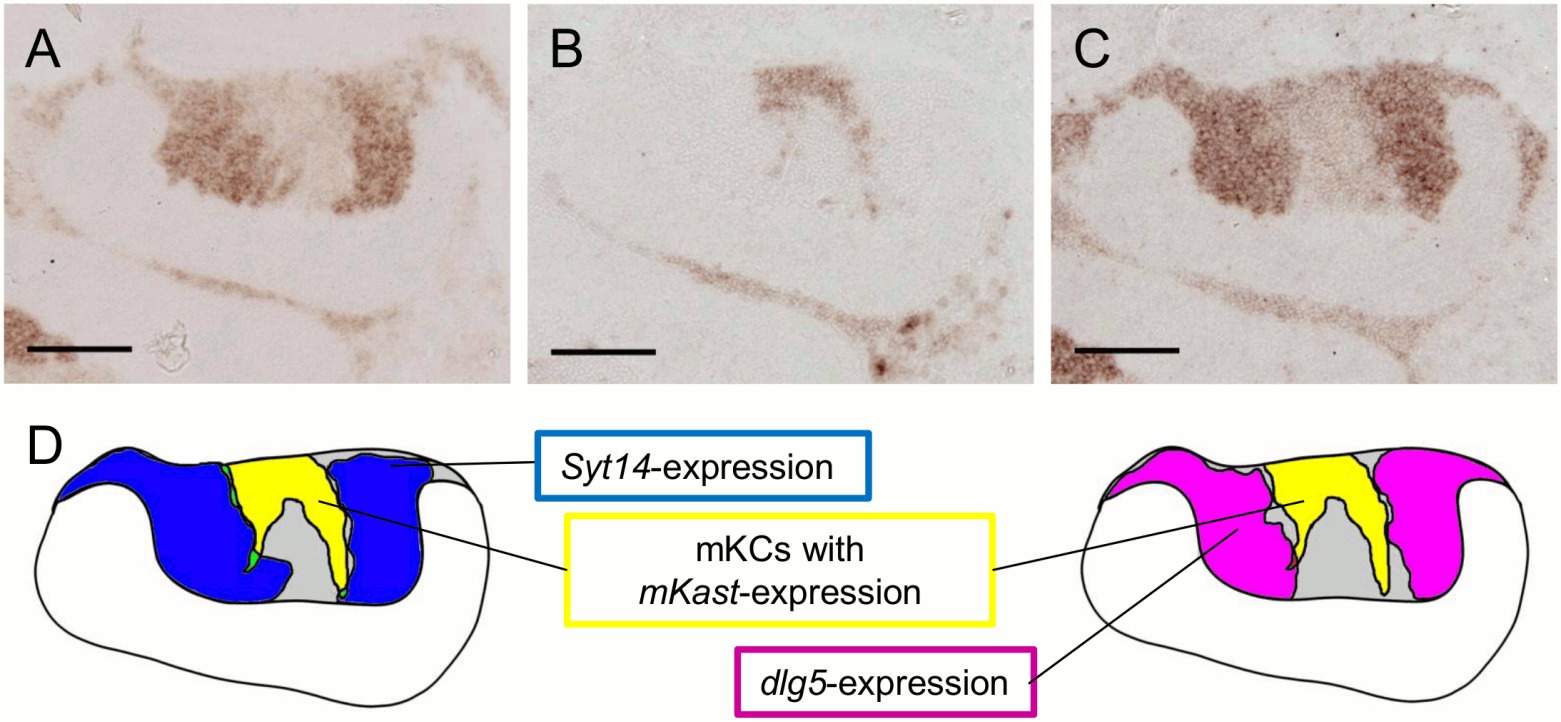
Expressions of *Syt14* and *dlg5* do not overlap with that of *mKast* in the worker MB. (A-C) *In situ* hybridization of *Syt14* (A), *mKast* (B) and *dlg5* (C) using serial worker MB sections. *mKast* signals represent the localization of mKC somata. (D) Schematic drawings of the expression of each gene in (A-C). Left illustration represents regions strongly expressing *Syt14* (blue) and *mKast* (yellow), respectively. Right illustration represents regions strongly expressing *dlg5* (magenta) and *mKast*, respectively. Note that regions strongly expressing *Syt14* and *dlg5* do not overlap with that strongly expressing *mKast*. Bars indicate 100μm. The probe for *dlg5* was prepared with the second set of primers mentioned in Materials and Methods.

Expression analysis of *Syt14* and *dlg5* suggested that they are preferentially expressed in the lKCs in the worker MBs. However, because the size of mKC somata is just intermediate of those of lKCs and sKCs [11], it is difficult to discriminate the boundary of mKCs and lKCs based on morphological observation. The mKCs are characterized with their preferential expression of *mKast* [11]. We therefore performed *in situ* hybridization for *Syt14*, *dlg5* and *mKast* with sequential sections of the worker brains to examine whether *Syt14* and *dlg5* are expressed only in the lKCs or in both the lKCs and mKCs. The brain regions preferentially expressing *Syt14* or *dlg5* did not overlap with that preferentially expressing *mKast* (Fig 7), indicating that both *Syt14* and *dlg5* were preferentially expressed in the lKCs but not in the mKCs.

### Quantitative expression analysis of *PLCe*, *Syt1*4, and *dlg5* in the developing brain

**Fig 8:**
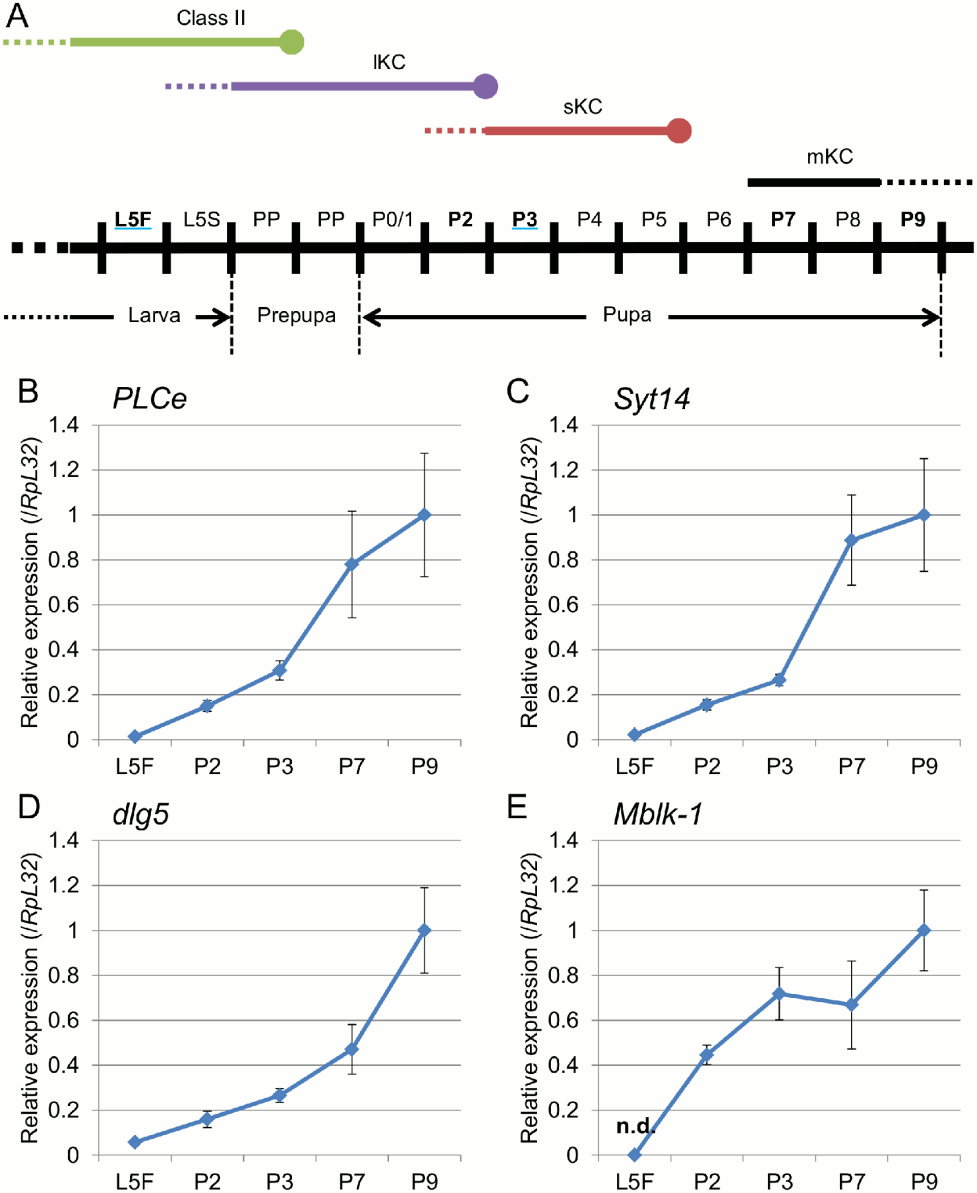
Quantitative RT-PCR of *PLCe*, *Syt14*, *dlg5*, and *Mblk-1* in the worker brain during metamorphosis. (A) Schematic drawing of the proliferation and differentiation of each KC subtype during the larval and pupal stages [32]. The lower bar indicates progression of the larval (L5F and L5S), prepupal (PP) and pupal stages (P0/1 to P9). The upper green, purple and red bars indicate the periods when the class-II KCs, lKCs, and sKCs are generated in the larval and pupal MBs. Black bar indicates the period when *mKast* expression is detected in the MBs. Dotted lines mean that the start points of proliferation of the class-II KCs, lKCs, and sKCs are obscure and that *mKast* expression was predicted but not analyzed previously [11]. L5F, fifth larval instar feeding stage; L5S, fifth larval instar spinning stage; PP, prepupa. (B-D) Change in the relative expression of *PLCe* (B), *Syt14* (C), *dlg5* (D), and *Mblk-1* (E) during metamorphosis, respectively. Points and bars on each developmental stage indicate average +- standard deviation. Expression level of each gene normalized to that of *RpL32* is indicated taking that at the P9 stage as 1. Three lots of samples were used for each developmental stage. n.d., not detected. For all genes, significant difference was detected by Kruskal-Wallis test (P < 0.05), whereas no significant difference was detected between expression levels at the adjacent stages (Steel-Dwass test, error rate was 0.05).

We next used quantitative RT-PCR to examine the expression levels of these genes in the larval (L5F) and developing pupal (P2, 3, 7, and 9) brains (Fig 8A). When we analyzed expression of two reference genes, *RpL32* and *gapdh*, expression of *RpL32* gradually decreased according to the progression of metamorphosis, whereas that of *gapdh* gradually increased (Fig S3A and S3B). In this experiment, we used *RpL32* as a reference gene to normalize expression levels of the above three genes. Relative expression levels of *PLCe*, *Syt14*, and *dlg5* prominently increased from the L5F to P9 stage for approximately 75-, 43-, and 18-folds, respectively (Fig 8B–8D), suggesting that they mainly function in the late pupal and adult brain. We previously showed that *Mblk-1* is preferentially expressed in the pupal lKCs [40]. Therefore, in the present study, we used *Mblk-1* as reference for the development of pupal MBs. While no significant expression of *Mblk-1* was detectable at the larval (L5F) stage, it increased prominently at the early pupal (P2) stage and reached a plateau at the P3 stage (Fig 8E), suggesting that, while Mblk-1 does not function in the larval brain, it plays roles in the pupal brains, as early as at the P2 stage. The larger variances of the relative expression of each gene at the P7/P9 stages may be due in part to the fact that only one bee per lot was used at these stages (Fig 8B–8E).

### *In situ* hybridization analysis of *PLCe*, *Syt14*, and *dlg5* in the larval and pupal brains

**Fig 9:**
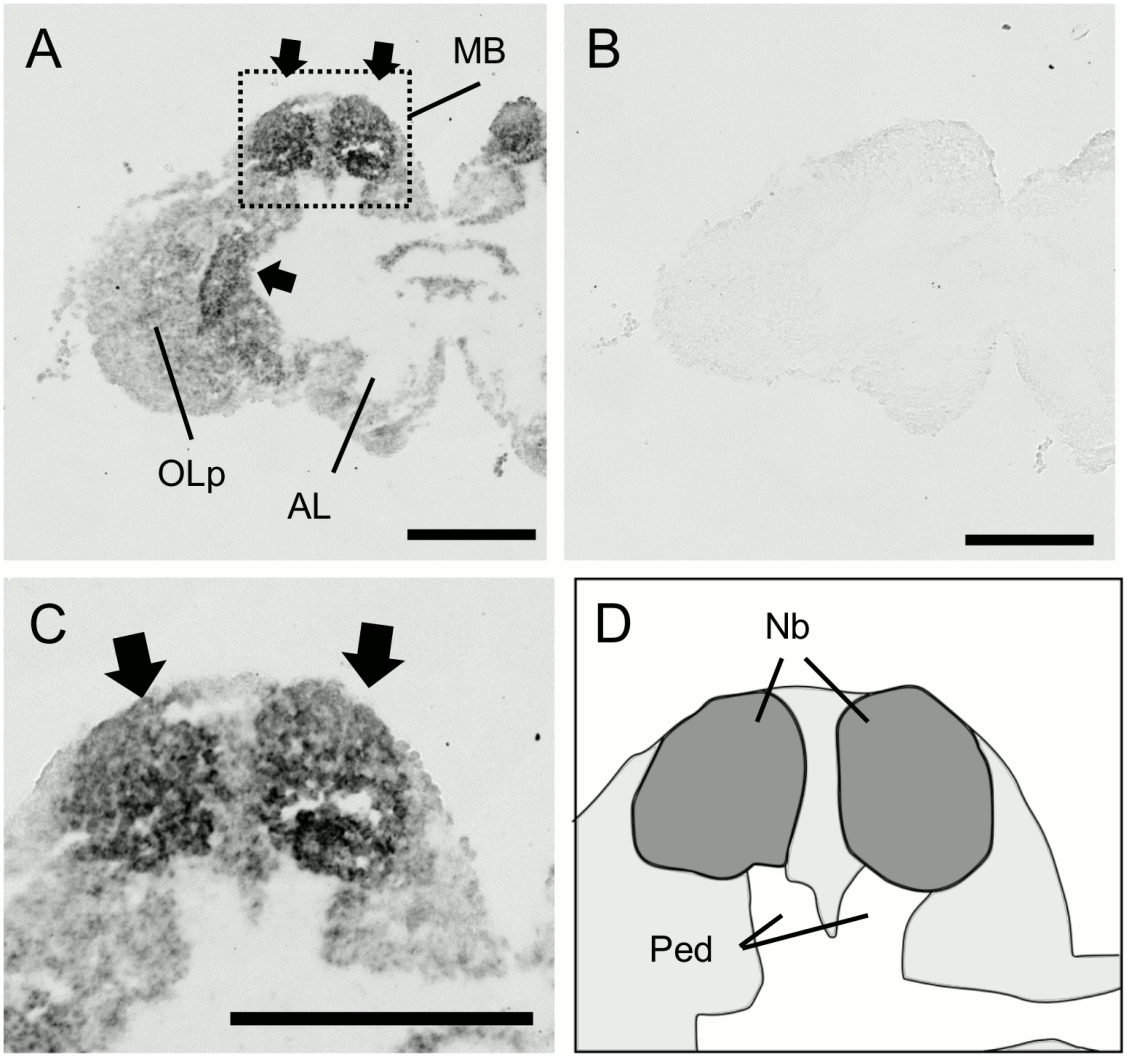
*In situ* hybridization of *RpL32* in the larval (L5F) brain. (A, B) Frontal sections of the left brain hemispheres hybridized with antisense (A) or sense (B) probes. Arrows indicate prominent signals in the MB neuroblasts and primordium of OL. (C) Magnified view of the MB shown by a square in (A). Arrows indicate prominent signals in the MB. (D) Schematic drawing of the brain structure seen in (C). Regions for the MB neuroblasts (Nb) and for the other cells are colored by grey and light grey, respectively. MB, mushroom body; OLp, optic lobe primordium; AL, antennal lobe; Ped, peduncles. Bars indicate 200μm.

Next we performed *in situ* hybridization analysis using sections of the larval (L5F) and pupal (P3) brains to examine how their expression profiles change during metamorphosis. We used the P3 stage pupae, because the lKCs are generated from the MB neuroblasts till the P2 stage (Fig 8A) [32] and their differentiation is expected to begin no later than around the P3 stage. We first analyzed expression of *RpL32* to discriminate brain areas where the cell bodies are located and examine general transcriptional activities in the larval (L5F) brain. Intense staining was detected in the whole brain with antisense probe, including the developing MBs and primordium of OLs [41] and ALs [35] as well as some cell bodies located near the central bodies (Fig 9A). No significant staining was detected with sense probe (Fig 9B), indicating that the signals were due to *RpL32* expression. Each MB of this stage comprises two neuroblast clusters and two peduncles that are located beneath the clusters, whereas calyces are not yet formed (Fig 9D) [32]. Relatively strong signals were detected in these MB neuroblast clusters (Fig 9C), possibly reflecting their enhanced protein synthesis.

**Fig 10:**
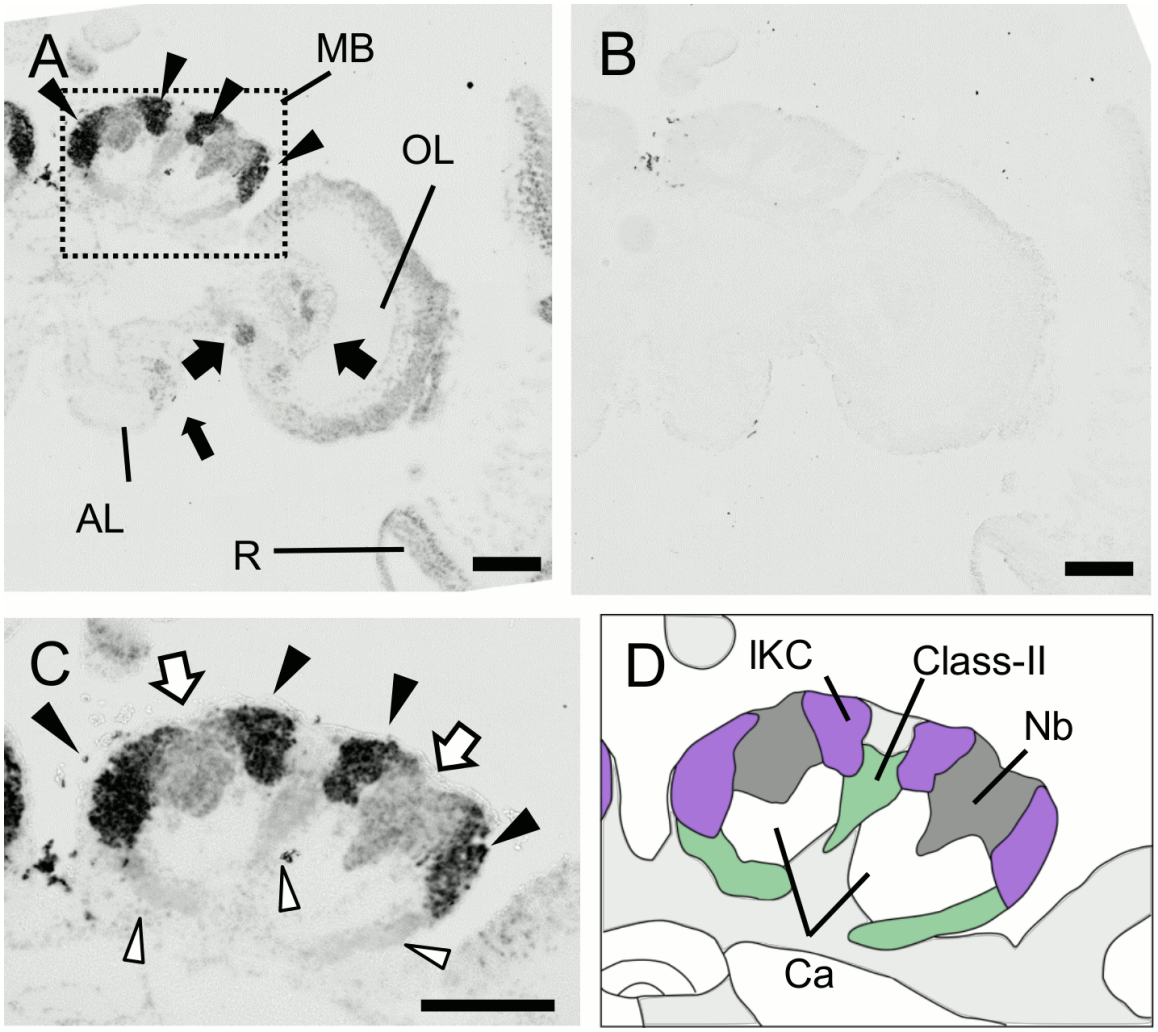
*In situ* hybridization of *Mblk-1* in the pupal (P3) brain. (A, B) Frontal sections of the right brain hemispheres hybridized with antisense (A) and sense (B) probes. Arrowheads and arrows indicate prominent signals in the MBs, OLs and ALs, respectively. MB, mushroom body; OL, optic lobe; AL, antennal lobe; R, retina. (C) Magnified view of the MB shown by a square in (A). Black and white arrowheads represent strong signals in the lKCs and weak signals in the class-II KCs, respectively. White arrows represent signals in the MB neuroblasts. (D) Schematic drawing of the brain structure seen in (C). The lKCs, class-II KCs, MB neuroblast clusters, and regions where somata of the other cells exist are colored by purple, green, grey, and light grey, respectively. Ca, calyces; Nb, MB neuroblasts; Ped, peduncles. Bars indicate 200μm.

We also analyzed expression of *Mblk-1* to discriminate brain regions where the lKC somata are located in the pupal (P3) brain. Strong signals were detected in the MBs with antisense probe, whereas weak signals were also detected in the whole brain (Fig 10A and 10B). Each MB of this stage comprises two neuroblast clusters, the sKCs that are localized at the both edges of neuroblast clusters, the lKCs that are localized sandwiched between the sKCs and class-II KCs and the class-II KCs that are localized at both edges of the MB peduncles (Fig 10D) [32]. The class-II KCs that are first generated by the neuroblast clusters are pushed away towards both edges of the MB peduncles, and the lKCs that are secondly generated are also pushed away around the neuroblast clusters [32]. In the pupal MBs, *Mblk-1* was expressed strongly in the lKCs and weakly in both the class-II KCs and neuroblast clusters (Fig 10C), coinciding with the previous report [40]. In the present study, the sKCs could not be discriminated based on the morphological observation, although they were discriminated based on expression of a dopamine receptor gene, Am*dop1*, in the preceding study [42].

**Fig 11:**
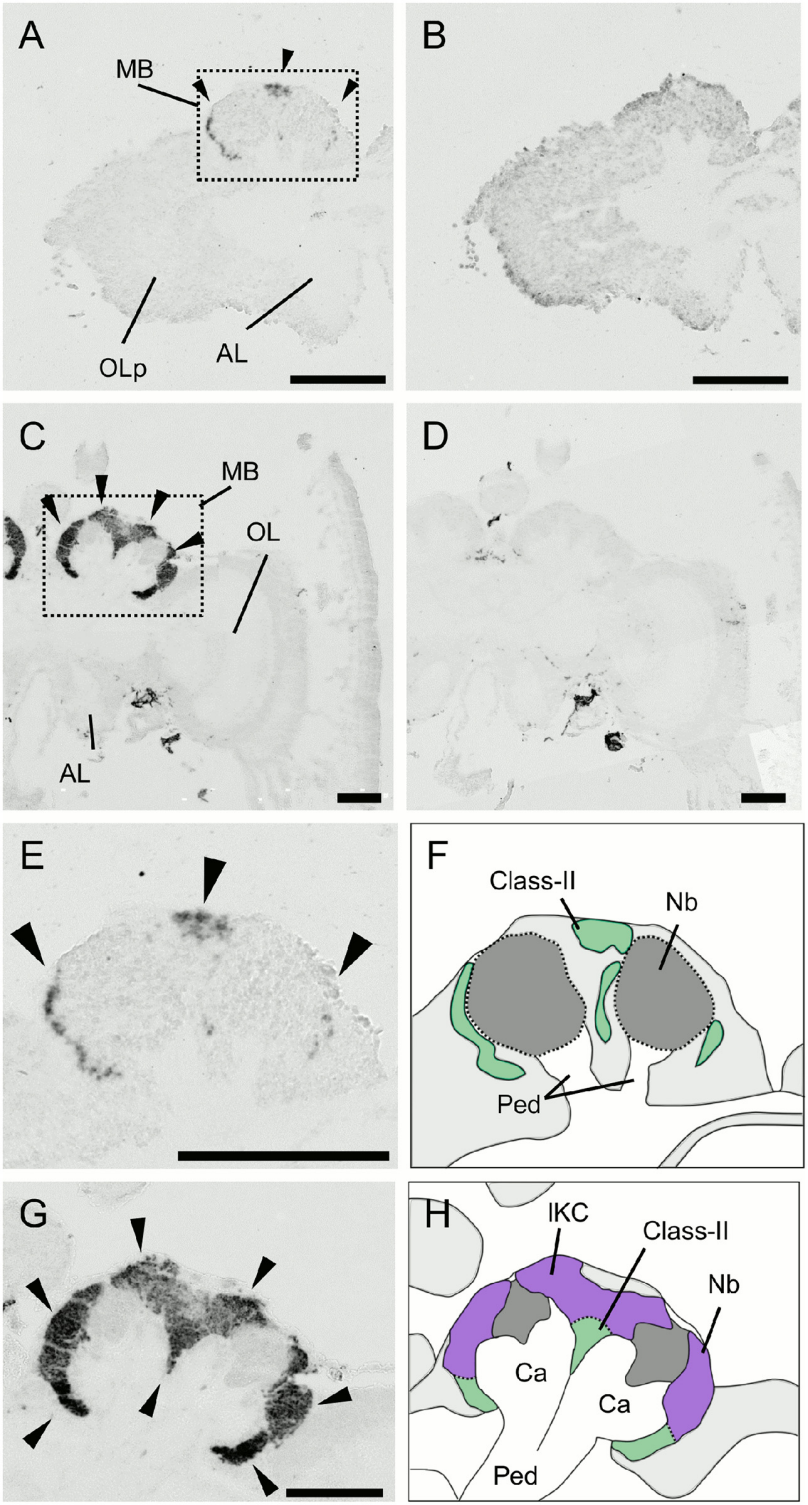
*In situ* hybridization of *PLCe* in the larval (L5F) and pupal (P3) brains. (A-D) Frontal sections of brain hemispheres of hybridized with antisense (A and C) and sense (B and D) probes. (A and B) The larval (L5F) brain left hemisphere. (C and D) The pupal (P3) brain right hemisphere. Arrowheads represent prominent signals in the MBs. MB, mushroom body; OLp, optic lobe primordium; OL, optic lobe; AL, antennal lobe. (E-H) Magnified views of the MBs shown in (A) and (C) and their schematic views. (E and F) The larval MBs. (G and H) The pupal MBs. Arrowheads represent prominent signals in the MBs. The lKCs, class-II KCs, MB neuroblast clusters, and regions where somata of the other cells exist are colored by purple, green, grey, and light grey, respectively. Ca, calyces; Nb, MB neuroblasts; Ped, peduncles. Bars indicate 200μm.

Then, we analyzed expression of *PLCe*, *Syt14*, and *dlg5* in the larval and pupal brain. Strong signals for *PLCe* were detected only in the MBs, while no significant expression was detected in any other brain regions in the larval brain (Fig 11A and 11B). In the MBs, strong signals were detected surrounding the MB neuroblast clusters, which correspond to the class-II KCs [32], but not in the neuroblast clusters (Fig 11E and 11F), indicating that *PLCe* is preferentially expressed in the class-II KCs in the larval (L5F) brain. In the pupal (P3) brain, strong signals were also detected only in the MBs, while no significant expression was detected in any other brain regions (Fig 11C and 11D). In the MBs, strong signals were detected surrounding the MB neuroblast clusters and peduncles, indicating that *PLCe* is preferentially expressed in both the class-II KCs and lKCs in the pupal (P3) brain (Fig 11G and 11H).

**Fig 12:**
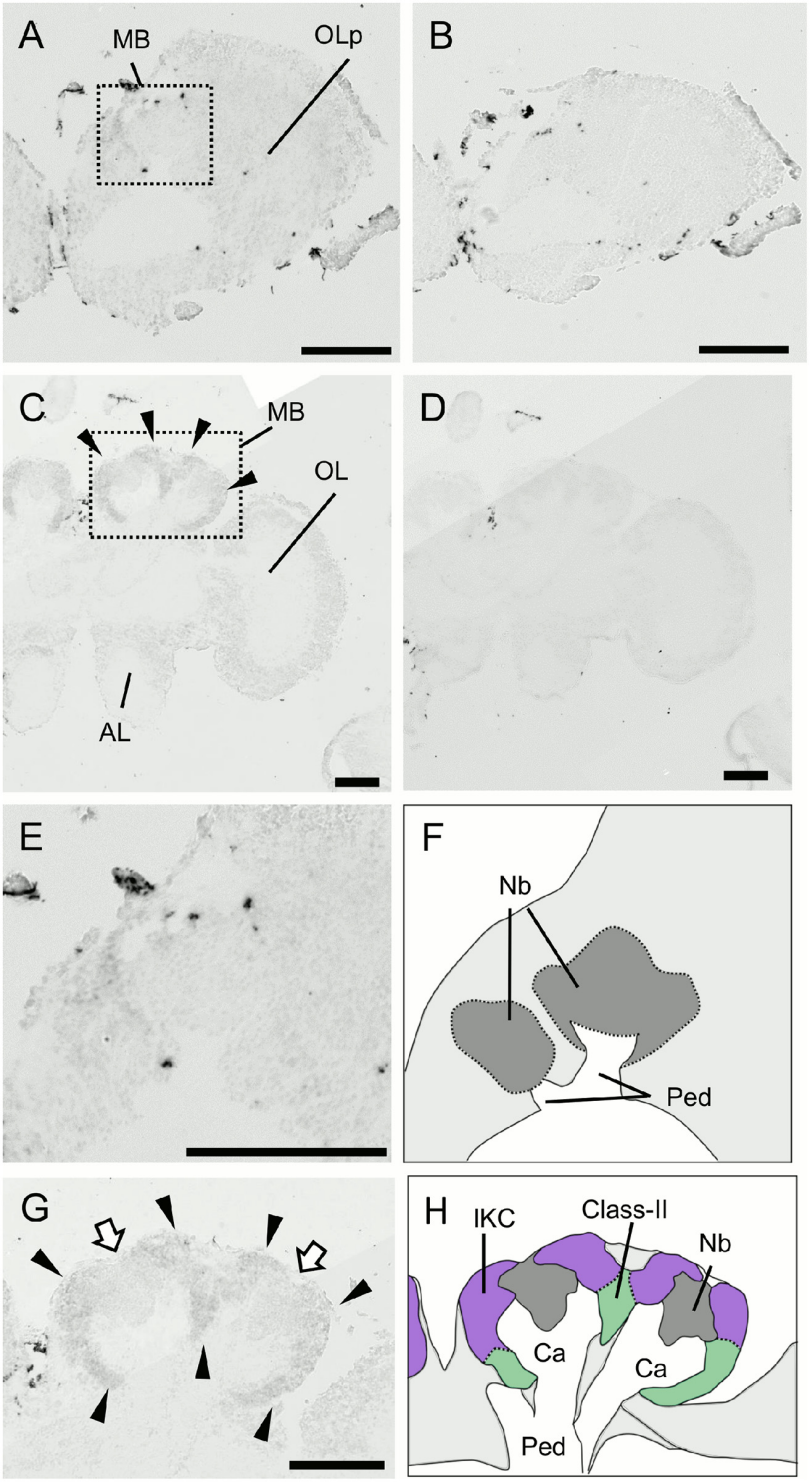
*In situ* hybridization of *Syt14* in the larval (L5F) and pupal (P3) brains. (A-D) Frontal sections of the brain right hemispheres hybridized with antisense (A, C) and sense (B, D) probes. (A, B) The larval (L5F) brain. (C, D) The pupal (P3) brain. Arrowheads in (C) indicate prominent signals in the MBs. MB, mushroom body; OLp, optic lobe primordium; OL, optic lobe; AL, antennal lobe. (E-H) Magnified views of the MBs shown in (A) and (C) and their schematic views. (E and F) The larval MBs. (G and H) The pupal MBs. Arrowheads indicate prominent signals in the lKCs and class-II KCs, while white arrows indicate weak signals in the MB neuroblast clusters. The lKCs, class-II KCs, MB neuroblast clusters, and regions where somata of the other cells exist are colored by purple, green, grey, and light grey, respectively. Ca, calyces; Nb, MB neuroblasts; Ped, peduncles. Bars indicate 200μm.

As for *Syt14*, no significant signal was detected in the larval (L5F) brain, including the MBs (Fig 12A, 12B, 12E and 12F), indicating that *Syt14* is not significantly expressed in the larval brain. In the pupal (P3) brain, moderate signals were detected in the MBs, while weak signals were detected in the whole brain (Fig 12C and 12D). In the MBs, significant signals were detected surrounding the MB neuroblast clusters and peduncles (Fig 12G and 12H), indicating that *Syt14* is preferentially expressed in both the class-II KCs and lKCs in the pupal brain.

**Fig 13:**
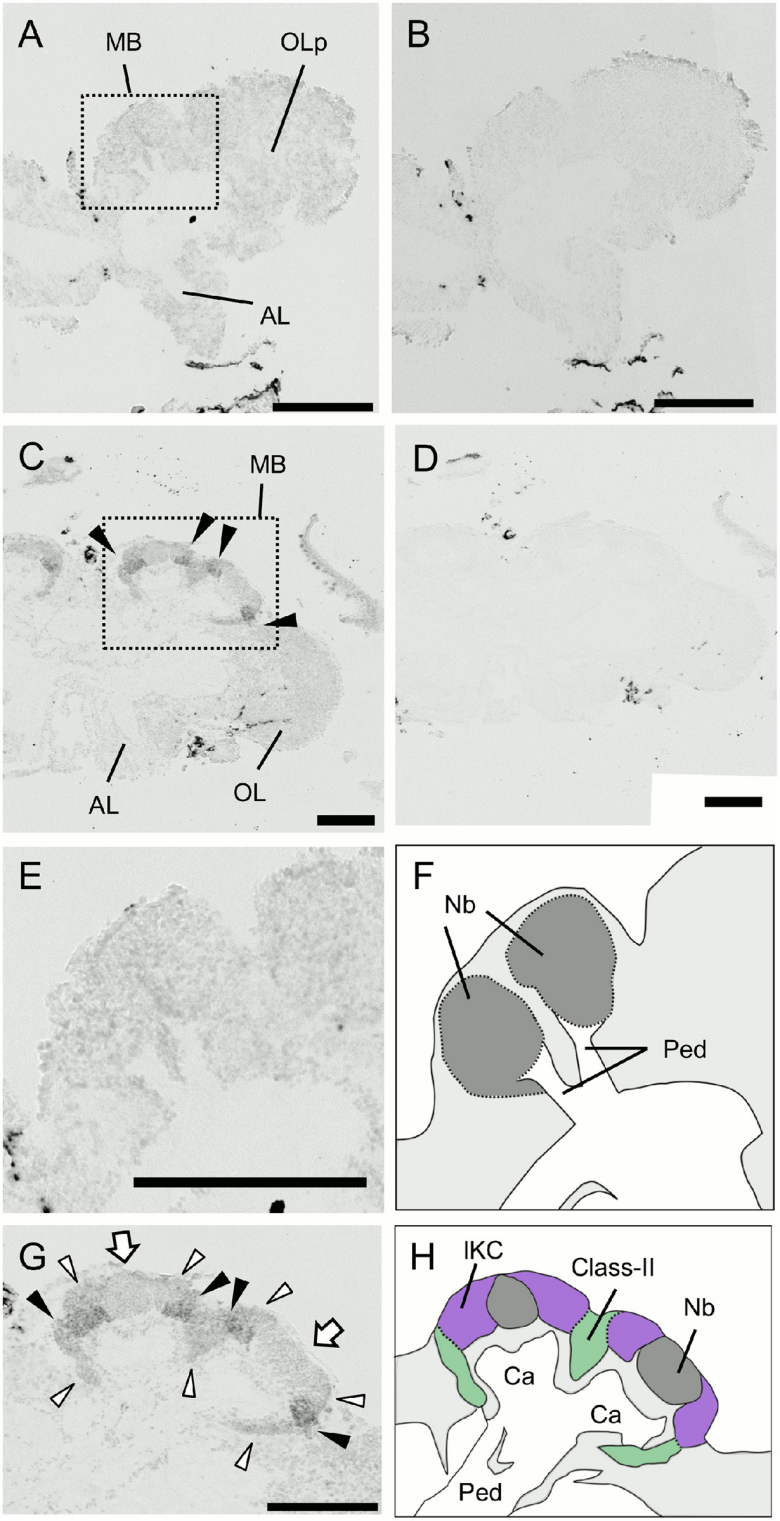
*In situ* hybridization of *dlg5* in the larval (L5F) and pupal (P3) brains. (A-D) Frontal sections of the brain right hemispheres hybridized with antisense (A and C) and sense (B and D) probes. (A, B) The larval (L5F) brain. (C, D) The pupal (P3) brain. Arrowheads in (C) indicate prominent signals in the MBs. MB, mushroom body; OLp, optic lobe primordium; OL, optic lobe; AL, antennal lobe. (E-H) Magnified views of the MBs shown in (A) and (C) and their schematic views. (E and F) The larval MBs. (G and H) The pupal MBs. Black and white arrowheads indicate moderate signals in the outer halves of the lKCs and weak signals in the remaining lKCs and class-II KCs, respectively. White arrows indicate weak signals in the MB neuroblast clusters. The lKCs, class-II KCs, MB neuroblast clusters, and regions where somata of the other cells exist are colored by purple, green, grey, and light grey, respectively. Ca, calyces; Nb, MB neuroblasts; Ped, peduncles. Bars indicate 200μm. The probes were prepared with the second set of primers mentioned in Materials and Methods.

**Fig 14:**
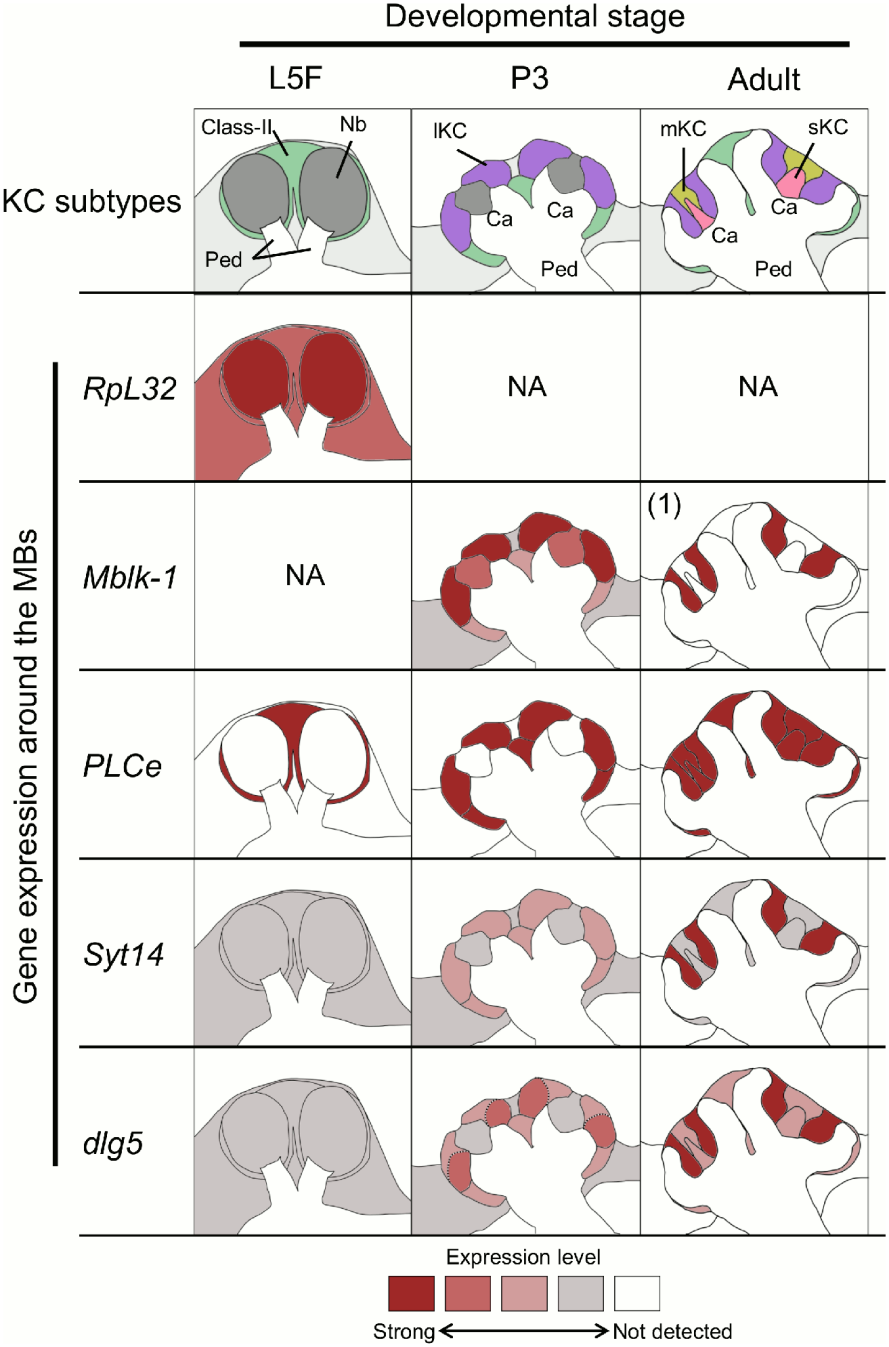
Summary of stage-specific gene expression profiles in the MBs during metamorphosis. Changes in the MB organization and gene expression profiles during metamorphosis are schematized. Expression level of each gene is represented by red intensity. NA, not analyzed in this study. (1) *Mblk-1* expression in the adult MBs reported previously [40] is also included in this figure. Ca, calyces; Nb, MB neuroblasts; Ped, peduncles.

As for *dlg5*, no significant signal was detected in the larval (L5F) brain, including the MBs (Fig 13A, 13B, 13E and 13F), indicating that *dlg5* is not significantly expressed in the larval brain. In the pupal (P3) brain, moderate signals were detected only in the MBs, while weak signals were detected in the other brain regions (Fig 13C and 13D). In the MBs, moderate signals were detected at the tips of the two developing calyces where parts of the lKC somata are localized (Fig 13G and 13H), indicating that *dlg5* is moderately expressed in the outer halves of regions where the lKCs somata exist. Developmental expression patterns of *PLCe*, *Syt14* and *dlg5* as well as *RpL32* and *Mblk-1* are summarized in Fig 14.

## Discussion

### Comprehensive identification of genes preferentially expressed in the honeybee MBs

In the present study, we used a cDNA microarray to newly identify 18 cDNA clones that are preferentially expressed in the honeybee MBs. *In situ* hybridization revealed that most of the clones identified are expressed in either an lKC-preferential or sKC and mKC-preferential manner (S1 Fig and S2 Table)., whereas none of them are expressed in an mKC-preferential manner, like *mKast* [11]. In addition, two clones (clones Nos. 28 and 539) were expressed preferentially in both the lKCs and sKCs but not in the mKCs, like *tachykinin-related peptide* [11, 43] and *juvenile hormone diol kinase* [11, 44], further supporting our notion that the mKCs have a unique gene expression profile in the honeybee MBs. While nearly half of the identified clones were mapped onto certain genes, enabling identification of the corresponding genes, some clones were mapped onto intergenic regions (S2 Table). This is probably due to the fact that the genome annotations are not yet precise and some genes may have transcriptional units that expand far upstream or downstream of the predicted ones.

Among the genes identified, *PLCe*, *Syt14* and *dlg5* were expressed preferentially in the MBs in the honeybee brain. Quantitative RT-PCR revealed that the expression levels of *PLCe*, *Syt14*, and *dlg5* in the MBs was approximately 31-, 6.8-, and 5.6-fold higher than those in the other brain regions (Fig 3). These levels are similar or higher when compared with *IP*_*3*_ *5-phosphatase* and *IP*_*3*_*R*, whose expression levels in the MBs are approximately 2.6- and 5.2-fold higher than that in the OLs, respectively [24] even though expression levels in brain regions were compared in a different way (for example, compared brain regions, and reference gene, etc.) between the previous and present studies. Besides the above three genes, one clone (clone No. 60) corresponded to the coding region of GB44412, which encodes a cell-cell adhesion molecule containing an immunoglobulin domain. n *Drosophila*, CG34113, a homolog to GB44412, is annotated as a gene involved in neural connectivity and its mutation affects the response to odorant [45]. It is plausible that the GB44412 product also regulates olfaction in the honeybee.

We could not find any orthologs for *PLCe*, *Syt14*, *dlg5* and GB44412 in the list of the genes whose expression is enriched in the MBs in the fruit fly [46], suggesting that preferential expression of these genes in the MBs is unique to certain Hymenopteran insects, including the honeybees. On the other hand, three clones (clone Nos. 231, 299, 440) identified in the present study belonged to the list of transcriptomic profile of the central nervous system, which was previously reported using three different honeybee species [47]. The brain regions where the genes are preferentially expressed, however, were not described in the preceding study [47].

### Preferential expression of *PLCe* in all KC subtypes, and *Syt14* and *dlg5* in the lKCs

In various animal species, PLCe plays a crucial role in calcium signaling as an enzyme hydrolyzing phosphatidylinositol 4,5-bisphospate to generate IP_3_ and diacylglycerol [48]. We previously demonstrated that expression of some of the genes for proteins involved in calcium signaling are enriched in the MBs, especially in the lKCs, in the honeybee brain. *IP*_*3*_*R* [23], *type I IP*_*3*_ *5-phosphatase* [24], *CaMKII* [25], *ryr*, and *reticulocalbin* [26] are preferentially expressed in the lKCs in the honeybee brain, whereas *protein kinase C* is expressed in all KC subtypes in the honeybee brain [25]. In mammals, IP_3_R and ryr are endoplasmic reticulum membrane proteins involved in IP_3_-mediated calcium release and calcium-induced calcium release, respectively [27] and type I IP_3_ 5-phosphatase is the major isoenzyme hydrolyzing IP_3_ [49]. Reticulocalbin is a calcium binding protein that acts to buffer calcium ions in the endoplasmic reticulum [50]. The MB-preferential *PLCe* expression further supports our previous notion that the expression of genes involved in calcium signaling is enriched in the honeybee MBs.

In mammals, synaptotagmin 1 (Syt1) is a major subtype of the synaptotagmin family that senses the calcium concentration in the presynapse to regulate synaptic transmission [51, 52]. Although *Syt14* might be responsible for some kind of neural diseases in humans [53, 54], the precise function of *Syt14* remains unknown. The lack of conserved amino acids in the calcium binding motif [51] and calcium-independent phospholipid binding [52, 53] suggest that Syt14 works in calcium independent membrane trafficking pathways in the lKCs.

In the embryonic mouse brain, dlg5 regulates localization of cadherin and spine formation [55]. In the honeybee, dendrite branching and length as well as the number of dendritic spines of some KCs in the ‘collar’ compartment of MB calyces dynamically increase according to the division of labor of workers [56]. Because both the lKCs and class-II KCs extend their dendrites into the collar compartment [5], it is plausible that *dlg5* is associated with the morphological changes and maintenance of the lKC dendritic spines.

### Change in *PLCe* expression pattern in the developing MBs during metamorphosis

Quantitative RT-PCR analysis revealed that relative expression levels of *PLCe*, *Syt14*, and *dlg5* constantly increased from the L5F to P9 stage, with prominent increases from the P3 to P7 stages for both *PLCe* and *Syt14* and from the P7 to P9 stages for *dlg5* (Fig 8B–8D). In contrast, expression of *Mblk-1* increased prominently from the L5F to P3 stages and reached a plateau at the P3 stage (Fig 8E). In metamorphosis of *Drosophila*, *E93*, which is an ortholog of honeybee *Mblk-1* and encodes a transcription factor, is required for apoptosis of the larval salivary gland downstream of ecdysone [57]. We previously showed that Mblk-1 is a sequence specific transcription factor and activates transcription of target genes in cultured mammalian cells [58, 59]. Taken together, it is plausible that expression of *Mblk-1* at the early pupal stages (L5F to P3) orchestrates subsequent induction of *Syt14* and *dlg5* in the lKCs at the later (P3 to P9) pupal stages.

*In situ* hybridization revealed distinct expression profiles of these three genes in the developing pupal MBs. Farris et al. (1999) reported that there are only the class-II KCs in the larval MBs based on the morphological observations [32]. Our results indicated that not only the larval class-II KCs but also differentiated KCs in the pupal brain preferentially express *PLCe* (Figs 11 and 14), demonstrating that preferential *PLCe* expression is a characteristic common to all KC subtypes rather than specific to the class-II KCs. Thus, *PLCe* could be a universal marker for labeling the entire honeybee MBs, irrespective to the developmental stage. On the other hand, no preferential expressions of *Syt14* and *dlg5* were detectable in the larval (L5F) MBs (Figs 12–14). It is not certain at present, however, whether the larval MBs do not preferentially express other genes that are expressed in an lKCs-preferential manner in the adult MBs.

### Change in *Syt14* and *dlg5* expression patterns in the developing MBs during metamorphosis

Although both *Syt14* and *dlg5* start to be expressed significantly in the pupal brains during metamorphosis, like *Mblk-1*, their developmental expression patterns considerably differed (Figs 8, 10 and 12–14). Moderate *Syt14* expression was detectable in both the class-II KCs and lKCs at the pupal (P3) stage, (Figs 12 and 14), suggesting that *Syt14* expression becomes more restricted to the lKCs at the later pupal stages. In contrast, although significant *dlg5* expression was detectable in both the class-II KCs and lKCs at the pupal (P3) stage, stronger *dlg5* expression was detected at the outer halves of the regions where the lKC somata exist (S1 Fig, Figs 3 and 14). Considering that the class-II KCs, lKCs and sKCs are sequentially generated by the MB neuroblasts and the early generated KCs are pushed away towards the outside of MB calyces at this pupal stage [32], it is plausible that the outer halves of the lKCs are more differentiated to express *dlg5*, while the inner halves of the lKCs are lesser differentiated and not ready to express *dlg5*.

Because *Syt14* is suggested to have a role in membrane trafficking [52, 53] and *dlg5* is involved in spine morphogenesis [55] in other animal species, it might be that the membrane trafficking and spine morphogenesis of the lKCs are fully established at the later pupal stages. The fact that there was no significant *Syt14* and *dlg5* expression in the larval brains (Figs 12–14) suggest that both membrane trafficking and spine morphogenesis are not well-developed in the larval class-II KCs, which coincides with the fact that the larval MBs lack calyces and have only thin peduncles (Figs 9, 10 and 14) [32, 60]. Considering that both *Syt14* and *dlg5* are not preferentially expressed in the adult *Drosophila* MBs [46], their preferential expression may be unique to the adult honeybee MBs.

### Comparison with known gene expression patterns in the larval and pupal honeybee MBs

Here, we compare gene expression patterns of *PLCe*, *Syt14*, and *dlg5*, with those of genes involved in neural functions and whose expression is relatively enriched in the MBs in the developing pupal or adult honeybee brains.

So far, expression of three dopamine receptor genes, Am*dop1*, Am*dop2* and Am*dop3*, in the pupal and adult honeybee brains has been investigated. Among them, Am*dop1* is widely expressed in the adult honeybee brain [61], whereas its expression in the MBs becomes more enriched in the sKCs in the forager brain [42]. In the pupal MBs, Am*dop1* is expressed in all differentiated KC subtypes with an intense expression in the newborn KC subtypes [42]. In addition, Am*dop1* is also expressed in certain brain regions located in the lateral protocerebrum adjacent to the developing OLs in the pupal brain [42]. Am*dop3* is also widely expressed in the pupal and adult brains [62]. As for Am*dop2*, while it is preferentially expressed in the sKCs in both the newborn worker and forager brains, it is also expressed in the class-II KCs, other brain regions, and glial cells in the pupal brain [42]. In the preceding study, Am*dop1* and Am*dop2* are suggested to be involved in growth and differentiation of various cells *via* the morphogenic role of dopamine in the developing pupal brain [42]. *In situ* hybridization using a probe corresponding to the coding region of Am*tyr1*, which encodes a honeybee tyramine receptor, revealed that Am*tyr1* is also widely expressed in the adult brain [63], whereas expression more enriched in the MBs is detectable with a probe corresponding to the 3’ UTR in the adult brain [64]. In the developing pupal brains, Am*tyr1* is expressed in all differentiated KC subtypes [64].

Compared to Am*dop1*, Am*dop3*, and Am*tyr1*, all of which are widely expressed in the adult honeybee brain, and in some brain regions including the MBs in the pupal brain, the honeybee *PLCe* is unique in that it is ubiquitously and preferentially expressed in all differentiated KC subtypes in all larval (L5), pupal (P3), and adult brains (Fig 14). On the contrary, compared to Am*dop2*, whose expression is enriched in the sKCs in the adult brain, *Syt14* and *dlg5* are unique in that they are preferentially expressed in the lKCs in the adult brain (Fig 14). Moreover, these two genes were expressed preferentially in the MBs in the pupal brain, unlike Am*dop2*.

Based on the developmentally regulated gene expression profiles of *Mblk-1*, *PLCe*, *Syt14*, and *dlg5*, differentiation of the KC subtypes in the pupal MBs is assumed to proceed as follows: ecdysone signaling first up-regulates *Mblk-1*, which subsequently induces preferential *dlg5* expressions in the outer halves of the differentiating lKCs at the pupal (P3) stage, and then preferential *Syt14* expression in the lKCs at the later pupal stages. On the other hand, the MB-preferential *PLCe* expression is maintained throughout the metamorphosis by other mechanisms, which seems common to all KC subtypes. We expect that our study provides not only insights into the differentiation of lKCs (*Syt14* and *dlg5*) but also molecular characteristics common to all KC subtypes (*PLCe*). We also expect that the list of genes with MB-preferential expression, including the above three genes, could contribute to our better understanding of the molecular and cellular bases underlying the higher brain functions of the honeybees.

## Supporting Information

S1 Fig *In situ* hybridization using cDNA clones (only antisense probe). OL, optic lobe; mCa, medial calyx; lCa, lateral calyx; AL, antennal lobe. The DIG-labeled RNA probes were detected using a DIG Nucleic Acid Detection Kit (Roche). The staining reaction was performed at 25°C for 18 h. *In situ* hybridization was repeated for two or three adults for each clone.
(PDF)

S2 Fig Quantitative RT-PCR analysis of *RpL32* as internal control gene in the adult brain. Expression levels in each brain regions were normalized by that in the other brain regions than the MBs. Bars indicate standard deviations.
(PDF)

S3 Fig Quantitative RT-PCR analysis of candidate internal control genes during metamorphosis. (A) *RpL32*, (B) *gapdh*. Points and bars on each developmental stage indicate average +- standard deviation. Expression levels were normalized against those on the stage P9 for each gene. For developmental stages, see Fig 8A.
(PDF)

S1 Table Clones detected positively in cDNA microarray and *in situ* hybridization. Positive clone numbers whose an MB-preferential expression was detected in microarray and confirmed by *in situ* hybridization. The signal intensity in microarray analysis is shown. Microarray was performed in duplicate. The average of signal intensity in the MBs was normalized by expression of *β-actin* and compared with that in the OLs and ALs. Clones were assigned as positive when they showed higher signals in the MBs than in the OLs or ALs by 2.0-folds or more (orange- or blue-colored cells, respectively). Then, their expression was further analyzed by *in situ* hybridization.Cy3(M), probes from the MBs; Cy5(O), probes from the OLs; Cy5(A); probes from the ALs.
(XLSX)

S2 Table Summary of genes identified by the differential display method and cDNA microarray. Expression level of each clone in each KC type was assigned to visual observation and represented semi-quantitatively using ‘+++’, ‘++’, ‘+’ or ‘-’. If the clone was located on a gene, the corresponding gene name was listed. When clones were mapped onto two overlapping genes or intergenic regions, corresponding genes were ‘not identified’. The gene corresponding to clone No. 523 was also not identified because this clone is not flanked by any genes. If any functions or domains are not assigned to the corresponding honeybee genes, they were referred to their predicted homologs in other animals registered in NCBI. 1): nucleotide sequences for these clones are represented in S3 Table.
(DOCX)

S3 Table Sequences of clone Nos. 60 and 495. Sequences not registered in NCBI database are represented here.
(DOCX)

S4 Table Proteins used in alignment. Accession number of proteins are referred from the NCBI database.
(XLSX)

S5 Table Identities between PLCe, Syt14, and dlg5 homologs. Identities (%) of amino acid sequences between *Apis mellifera* and other organisms were analyzed using the ClustalW multiple alignment program. Dm, *Drosophila melanogaster*; Tc, *Tribolium castaneum*; Ce, *Caenorhabditis elegans*; Aa, *Aedes aegypti*; Cq, *Culex quinquefasciatus*; Mm, *Mus musculus*; Hs, *Homo sapiens*. (A) Results of PLCe, whose homolog is not annotated in the fruit fly. (B) Syt14 and Syt16 results. On the genome of *Tribolium castaneum*, Syt16, but not Syt14, is annotated. (C) dlg5 results. NK; nucleoside/nucleotide kinase domain. Note that 1) domains are not annotated, although the sequence has been identified, 2) domains are annotated as C2B_Synaptotagmin-14_16 instead of C2, and 3) guanylate kinase domain was annotated within the guanylate kinase homolog domain. Identity between the NK domain and the guanylate kinase domain was higher (42.3%, as shown in the table) than that calculated using the guanylate kinase homology domain (41%).
(XLSX)
