# Supplementary material for: Analysis of the Differentiation of Kenyon Cell Subtypes Using Three Mushroom Body-Preferential Genes during Metamorphosis in the Honeybee (Apis mellifera L.)
Source: PLoS One. 2016 Jun 28;11(6):e0157841. doi: 10.1371/journal.pone.0157841 (PMC4924639; doi:10.1371/journal.pone.0157841)
Supplement: S1 Fig — OL, optic lobe; mCa, medial calyx; lCa, lateral calyx; AL, antennal lobe. The DIG-labeled RNA probes were detected using a DIG Nucleic Acid Detection Kit (Roche). The staining reaction was performed at 25°C for 18 h. In situ hybridization was repeated for two or three adults for each clone. (PDF) [file pone.0157841.s001.pdf]

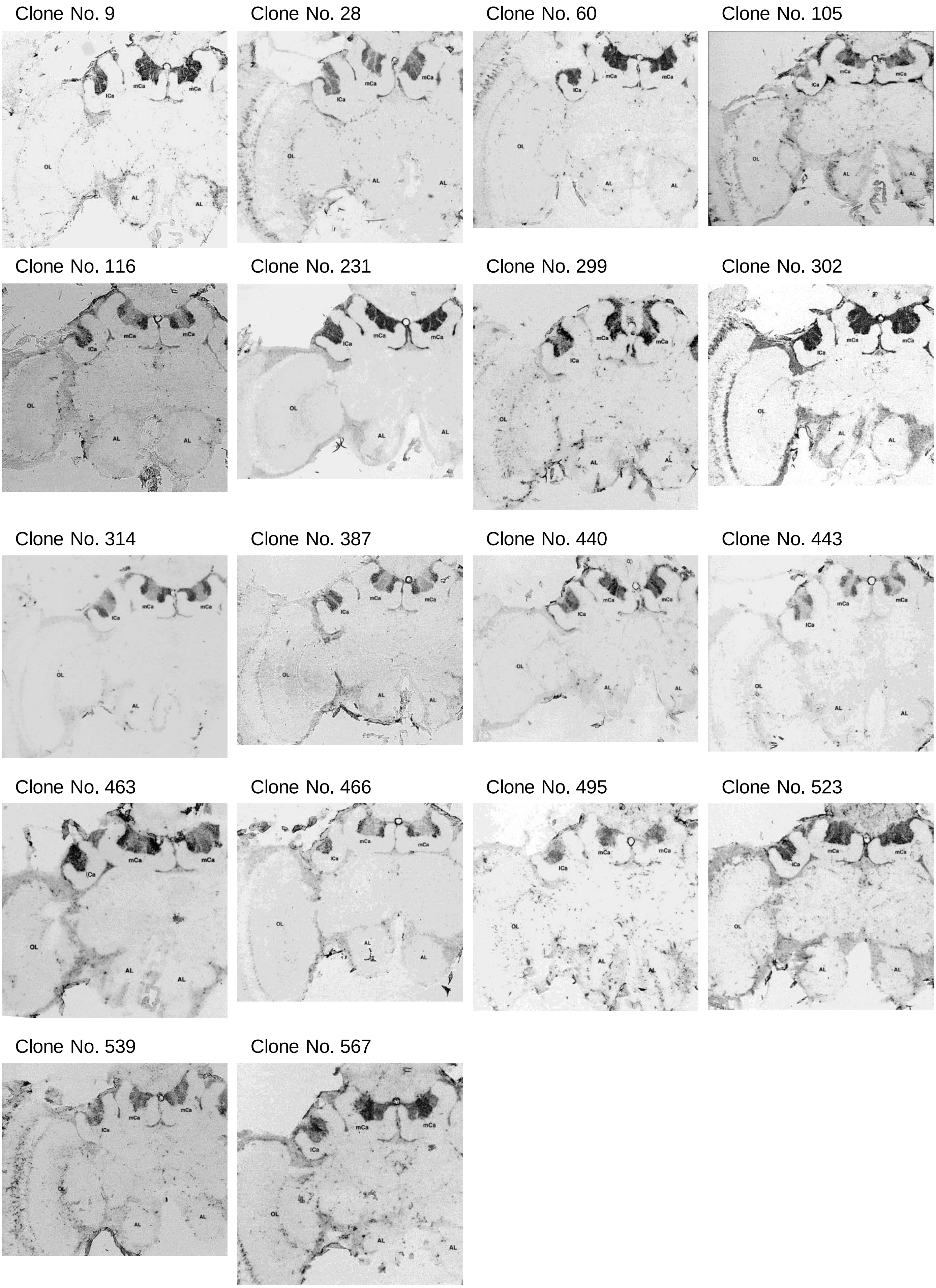
Clone No. 9
Clone No. 28
Clone No. 60
Clone No. 105
Clone No. 116
Clone No. 231
Clone No. 299
Clone No. 302
Clone No. 314
Clone No. 387
Clone No. 440
Clone No. 443
Clone No. 463
Clone No. 466
Clone No. 495
Clone No. 523
Clone No. 539
Clone No. 567

S1 Fig
