# Supplementary figures and images for: Analysis of the Differentiation of Kenyon Cell Subtypes Using Three Mushroom Body-Preferential Genes during Metamorphosis in the Honeybee (Apis mellifera L.)
Source: PLoS One. 2016 Jun 28;11(6):e0157841. doi: 10.1371/journal.pone.0157841 (PMC4924639; doi:10.1371/journal.pone.0157841)

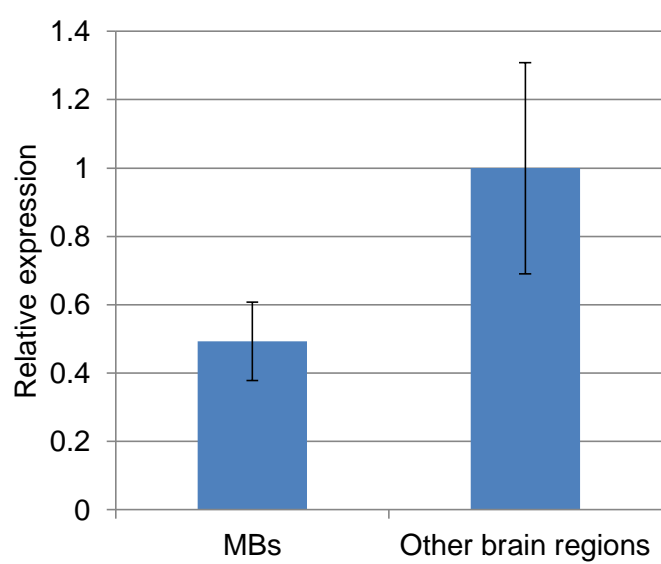

Supplement: S2 Fig — Expression levels in each brain regions were normalized by that in the other brain regions than the MBs. Bars indicate standard deviations. (PDF) [file pone.0157841.s002.pdf]

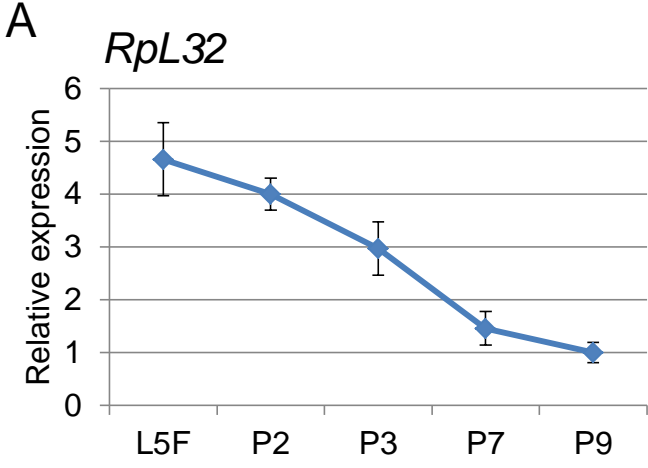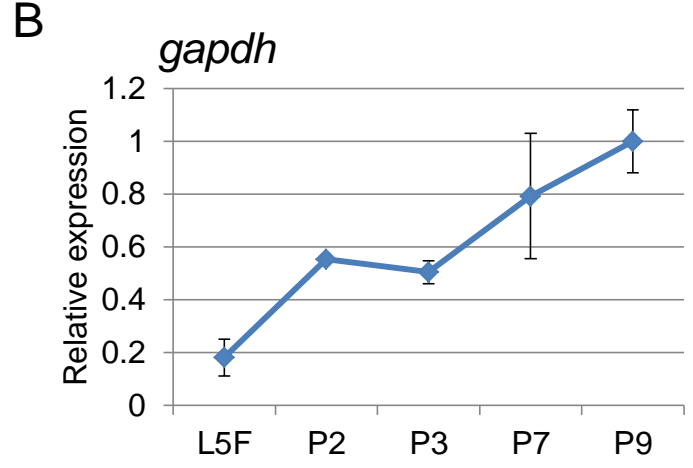

Supplement: S3 Fig — (A) RpL32, (B) gapdh. Points and bars on each developmental stage indicate average +- standard deviation. Expression levels were normalized against those on the stage P9 for each gene. For developmental stages, see Fig 8A. (PDF) [file pone.0157841.s003.pdf]
