# Supplementary material for: Analysis of the Differentiation of Kenyon Cell Subtypes Using Three Mushroom Body-Preferential Genes during Metamorphosis in the Honeybee (Apis mellifera L.)
Source: PLoS One. 2016 Jun 28;11(6):e0157841. doi: 10.1371/journal.pone.0157841 (PMC4924639; doi:10.1371/journal.pone.0157841)
Supplement: S2 Table — Expression level of each clone in each KC type was assigned to visual observation and represented semi-quantitatively using ‘+++’, ‘++’, ‘+’ or ‘-’. If the clone was located on a gene, the corresponding gene name was listed. When clones were mapped onto two overlapping genes or intergenic regions, corresponding genes were ‘not identified’. The gene corresponding to clone No. 523 was also not identified because this clone is not flanked by any genes. If any functions or domains are not assigned to the corresponding honeybee genes, they were referred to their predicted homologs in other animals registered in NCBI. 1): nucleotide sequences for these clones are represented in S3 Table. (DOCX) [file pone.0157841.s005.docx]

S2 Table. Summary of genes identified by the differential display method and cDNA microarray.

Clone Nos. 　Accession Nos in GenBank Expression levels in Predicted honeybee gene　　　Function or protein domain Position of probes

　　　　　lKC/ mKC/ sKC/ class-II KC

Intracellular signal transduction

No.105 　BP538211.1 　　　+/ ++/ ++/+++ LOC410870/GB49753 　　　otopetrin Intron

No.116 　AB811436.1 　　　+++/ +/ +/ + LOC412965/GB48632 　　　C2Asynaptotagmin domain 3’UTR

No.231 　BP539402.1 　　　+++/+++/+++/+++ LOC408804/GB42984 　　　Phospholipase C 3’UTR

No.314 　BP539538.1 　　　+++/ +/ +/ + LOC410178/ GB48789　　　PDZ domain Coding region

Transcription factors

No.440 　BP538668.1 　　　++/+++/+++/+++ LOC411009/ GB44041　　　DNA binding domain 3’UTR

No.567 　BP538879.1 　　　++/+++/+++/ ++ LOC409022/GB17617 　　　BTB domain 3’UTR

Neurotransmitter receptor

No.28 　BP538086.1 　　　+++/ -/ ++/ ++ LOC551680/GB15968　　　Histamine gated chloride channel 3’UTR

Cell - cell adhesion molecules

No.60 　^1)^+++/+++/+++/+++ LOC413370/GB44412 　　　Immunoglobulin domain Coding region

ncRNA

No.9 　BP538139.1 　　　+++/+++/+++/+++ LOC102655374 　　　ncRNA 2983bp upstream

(No genes annotated 5’ of cDNA clone)

No.539 　BP538837.1 　　　+++/ +/+++/+++ LOC102655642 　　　ncRNA Overlapped

Unknown function

No.299 　BP539504.1 　　　+++/ ++/ ++/ ++ LOC408988/GB53322 　　　Similar with Mouse GM323 　　　61bp overlapped with 3’UTR

Not identified

No.302 　BP539452.1 　　　+++/+++/+++/+++ LOC408951/ GB42560　　　14-3-3 epsilon 1771bp downstream of 3’UTR

LOC725708/GB42527　　　uncharacterized 802bp downstream of 3’UTR

No.387 　BP538491.1 　　　+++/ +/ +/ ++ LOC551623/GB42292 　　　RNA recognition motif 3’UTR

LOC102655730 　　　ncRNA Overlapped

No.443 　BP538686.1 　　　+++/ +/ +/ + LOC551098/ GB45335　　　Immunoglobulin domain 7573bp downstream of 3’UTR

LOC100576838/GB45336　　trypsin 534bp upstream of 5’UTR

No.463 　BP538730.1 　　　+++/ ++/ ++/ ++ LOC102654776 　　　Paired Box 1168bp downstream of 3’UTR

LOC408886/GB41430　　　Transmembrane protein 4460bp downstream of 3’UTR

No.466 　BP538761.1 　　　+++/ +/ +/ + LOC725970/GB53305　　　GDP-GTP exchangers 2797bp downstream of 3’UTR

LOC408992/GB53306 　　　sterol sensing domain 3413bp upstream of 5’ UTR

No.495 　^1)^+/ ++/ ++/ ++ LOC551010/GB43416 　　　Acetylcholine receptor 7384bp downstream of 3’UTR

LOC550962/GB43487 　　　exonuclease 10636bp downstream of 3’ UTR

No. 523 　BP538810.1 　　　+++/+++/+++/+++ Not found 　　　Not found Not flanked by annotated genes
