# Supplementary material for: Analysis of the Differentiation of Kenyon Cell Subtypes Using Three Mushroom Body-Preferential Genes during Metamorphosis in the Honeybee (Apis mellifera L.)
Source: PLoS One. 2016 Jun 28;11(6):e0157841. doi: 10.1371/journal.pone.0157841 (PMC4924639; doi:10.1371/journal.pone.0157841)
Supplement: S3 Table — Sequences not registered in NCBI database are represented here. (DOCX) [file pone.0157841.s006.docx]

S3 Table. Sequences of clone Nos. 60,495.

No. 60

GCCGCGGGAATTCGATTGTTTTTTTTTTGCGGTGATTGTATGATATCTGGCTGATTATTGAGCGCATAATCAGTAACTTGTTTTAATGTATATTCAACGAAATAAGTATGATCTCCGTGTGTGACATCAAGCAGAA

No. 495

AGCTTCTGCTTGATGAACAGAGGAGCCTGATAACGGAAATGACGANNCTTAACGTGGAAATTGCCAATCGTCGTCTAATTTATCGGGTTGCAGCCAATTTCGACTTGATATCGAAGATCCACAGCTCGAGATTCGATACTGCACGTGCTTGGAACGCTATCGACAATCCGGAAATCTTTCATTGTAGCTTCTATTAGAGATTCCATAACTAATAGGAGGAAAAGTAGACGACGTGCATTCTTGTTTTGATTTGTGTCATGATTTATATCGTTTATCTCGAGAACGAAAATTGCTCAAAATTGATCAAACCAATAATCAAGTCAATATTGTGGAACTTTTTGGAAAAAGAAGTGAGAAAAANATTATGAATTATANAAGATGTTAGTTTTTGAATATAATTTAAAAGTAATTAACGGTTGTTGCAATAAAAATGTGTTATGTATTAGCACACNCGNATGATTAATTAATGATGATTTACTGGAAAAAAAAAA
